# A novel multi objective grey wolf optimization fuzzy miner for process discovery: Incorporating robustness and explainability in model evaluation

**DOI:** 10.1371/journal.pone.0343119

**Published:** 2026-03-04

**Authors:** Mohammad Salehi, Rauof Khayami, Mirpouya Mirmozaffari

**Affiliations:** 1 Computer Engineering and Information Technology Department, Shiraz University of Technology, Shiraz, Iran; 2 Department of Industrial Engineering, Faculty of Industrial and Mechanical Engineering, Qazvin Branch, Islamic Azad University, Qazvin, Iran; Istinye University: Istinye Universitesi, TÜRKIYE

## Abstract

Process mining provides methodologies for analyzing, monitoring, and improving processes based on event logs. This study introduces Fuzzy Multi-Objective Grey Wolf Optimization (Fuzzy MOGWO), which integrates fuzzy modeling with a multi-criteria metaheuristic optimization approach. The proposed framework simultaneously optimizes six metrics: Fitness, Precision, Generalization, Simplicity, Robustness, and Explainability with the latter two newly proposed to evaluate noise resilience and analyst interpretability. A normalized scoring mechanism, based on the L₂ norm of all metrics, ensures balanced evaluation across objectives. Fuzzy MOGWO is benchmarked against Alpha Miner, Inductive Miner, and Fuzzy Miner using 10 synthetic noise-free logs, 10 synthetic noisy logs with 5–20% injected noise, and 3 real-world logs. Under noise-free conditions, it achieved a normalized score of 0.329, surpassing the best baseline (0.288) by 14.24%. In noisy environments, its score (0.440) exceeded the top competitor (0.378) by 16.40%. On real-world logs, it outperformed competitors in 4 out of 6 metrics, compared to 2 out of 6 for the PSO-based miner. These results demonstrate substantially improved effectiveness, robust performance in the presence of noise, and enhanced interpretability, establishing Fuzzy MOGWO as a comprehensive and reliable solution for challenging process discovery tasks.

## 1. Introduction

In today’s dynamic digital era, organizations increasingly turn to data-driven strategies to understand, oversee, and refine their operational workflows [1]. A process, at its core, is a purposeful sequence of tasks designed to achieve specific business goals. Historically, analyzing these workflows relied on subjective evaluations or labor-intensive manual records, often leading to inefficiencies and errors [2]. The rise of process mining has transformed this landscape, offering a powerful way to uncover meaningful insights directly from the event logs produced by modern information systems, enabling businesses to operate with greater clarity and precision [3].

Process mining serves as a vital bridge between data science and business process management, empowering organizations to reveal, monitor, and enhance real-world processes through the analysis of event logs. This field encompasses three key practices: process discovery, which builds a process model from event logs without prior assumptions; Conformance Checking, which evaluates how well a discovered model aligns with an existing one to pinpoint discrepancies; and Enhancement, which refines a process model by incorporating insights from actual process executions. Together, these practices provide a robust framework for understanding and improving operational efficiency, grounded in the data that organizations already generate [1].

The IEEE Task Force on Process Mining, a leading authority in shaping this discipline, underscores the importance of transparency, reproducibility, and interpretability in process discovery. It has outlined 11 foundational challenges that define the future of this emerging field. Among these, a critical need exists for innovative ways to assess the quality of process discovery algorithms [4]. The task force identifies four essential quality dimensions for evaluating process models: Consistency, which measures how accurately a model reflects the behaviors captured in the event logs; Precision, which ensures the model does not overgeneralize to unintended behaviors; Generalization, which gauges the model’s ability to adapt to new, plausible behaviors based on current data; and Simplicity, which reflects the model’s structural clarity and ease of understanding. These dimensions guide the development of more effective and reliable process discovery methods, addressing the evolving needs of organizations in a data-driven world [1].

Despite their widespread adoption, these four quality dimensions are known to exhibit inherent trade-offs, where improvements in one criterion may adversely affect others. Moreover, when applied to noisy or incomplete real-world event logs, they may not fully capture the structural and behavioral complexity of discovered models. This limitation has motivated recent research toward richer evaluation perspectives that better reflect process variability and real operational conditions [2].

While pioneering algorithms like Alpha Miner [3], Heuristic Miner [5], Fuzzy Miner [6], Flexible Heuristics Miner [7], and Inductive Miner [8], have introduced meaningful improvements in flexibility and precision, these methods continue to grapple with fundamental limitations. Specifically, they fail to achieve robust multi-objective optimization across all quality dimensions and struggle to scale effectively to meet the demands of complex, noisy, and high-variance real-world event logs. These shortcomings underscore the necessity for advanced optimization approaches.

To address these challenges, scholars have increasingly embraced computational intelligence techniques, particularly evolutionary algorithms and swarm intelligence. These approaches excel at reconciling conflicting performance indicators, fostering a balanced and effective process model. Drawing inspiration from the social hierarchy and cooperative hunting behaviors of Grey Wolves, the Grey Wolf Optimization (GWO) [9] algorithm offers a robust framework for optimization. In this research, we present an advanced iteration of the multi-objective Grey Wolf Optimization algorithm [10,11], termed Fuzzy MOGWO, and designed to concurrently optimize multiple competing objectives. This algorithm incorporates an external Pareto-based archive and employs adaptive leadership strategies to explore diverse trade-offs within the solution space, paving the way for more nuanced and effective process discovery.

Our proposed approach represents process models as fuzzy causal matrices, which are evaluated through an enhanced fitness function encompassing six quality criteria: the four traditional IEEE metrics Consistency, Precision, Generalization, and Simplicity alongside two newly introduced structural indices, Robustness and Explainability. This comprehensive evaluation ensures that models not only align with observed behaviors but also withstand noise and remain interpretable to users. By integrating fuzzy logic, the method captures the subtle dynamics of processes, offering a more flexible and realistic representation of real-world workflows.

Recent advances in process discovery algorithms reveal a persistent gap between theoretical models and practical applications, particularly when dealing with noisy, incomplete, and large-scale event logs. While classical techniques such as Alpha Miner, Heuristic Miner, and Inductive Miner perform well on structured logs, their effectiveness diminishes under real-world uncertainty. Although evolutionary and fuzzy-logic-based approaches have improved flexibility and optimization capabilities, existing methods still lack a unified framework that simultaneously addresses robustness, explainability, and multi-objective model quality in a measurable and systematic manner.

This study addresses these critical deficiencies by integrating a Fuzzy Causal Matrix representation with a multi-objective Grey Wolf Optimization scheme, simultaneously optimizing six quality metrics: Precision, Generalization, Consistency, Simplicity, Robustness, and Explainability. In doing so, the proposed Fuzzy MOGWO framework not only bridges the methodological gap between Robustness and interpretability but also provides a reproducible, statistically validated benchmark for real-world process discovery. The review in Section 2 systematically identifies these limitations across state-of-the-art approaches, directly motivating the objectives and design of the proposed method.

This study contributes four significant advancements to the field of Process Mining. First, it develops six fitness functions tailored to the six performance evaluation criteria, enabling their simultaneous optimization. Second, it encodes each wolf in the optimization process as a Fuzzy Causal Matrix, a candidate process model where each element represents the membership value of the connection between process activities, thereby enhancing the model’s ability to reflect complex process behaviors. Third, the algorithm leverages Mamdani fuzzy rules to dynamically define and update the α, β, and δ positions in each iteration, guiding the search with a sigmoid function to ensure precise navigation of the solution space. Finally, it employs a normalized scoring mechanism to identify the optimal process model from the Pareto front, delivering a balanced and high-quality solution. The efficacy of this approach is demonstrated through evaluations on 20 synthetic event logs (both noisy and noise-free) and three real-world event logs, benchmarked against established algorithms such as Alpha Miner, Inductive Miner, and Fuzzy Miner.

**Fig 1:**
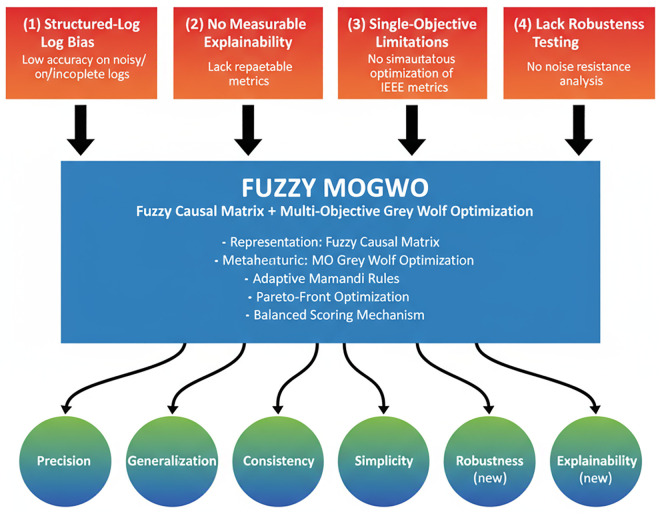
Structure of Fuzzy MOGWO algorithm in Process Mining, considering six metrics.

Fig 1 illustrates how the proposed Fuzzy MOGWO framework directly addresses the critical research gaps identified in the state-of-the-art analysis. Current methods suffer from limitations such as structured-log bias, lack of measurable Explainability, single-objective optimization constraints, and insufficient Robustness testing. Our approach integrates a Fuzzy Causal Matrix representation with Multi-objective Grey Wolf Optimization, enabling simultaneous improvement across six IEEE-recognized quality dimensions: Precision, Generalization, Consistency, Simplicity, Robustness, and Explainability. This visual mapping links the identified gaps to the study’s objectives, highlighting the novelty and practical relevance of the proposed method.

Building upon the above analysis, the present research pursues four specific objectives:

(1) To design a Fuzzy Causal Matrix representation capable of capturing complex, uncertain, and concurrent process behaviors.
(2) To define and integrate measurable Robustness and Explainability metrics alongside the four IEEE quality dimensions, enabling multi-faceted evaluation under both noise-free and noisy conditions.
(3) To develop and implement a multi-objective Grey Wolf Optimization scheme that simultaneously optimizes all six metrics, ensuring a balanced trade-off in model quality.
(4) To validate the proposed Fuzzy MOGWO framework on a broad range of synthetic and real-world event logs, demonstrating its practical relevance and superiority over state-of-the-art baselines.

These goals are directly motivated by the research gaps highlighted in Fig 1 and are further linked to real-world applicability in domains such as healthcare, manufacturing, and service operations where interpretability and noise resilience are critical requirements.

### 1.1. Scientific relevance

The growing complexity, variability, and uncertainty of modern information systems have exposed fundamental limitations in traditional process discovery methods, increasing the scientific importance of robust and interpretable modeling approaches. Event logs from domains such as healthcare, financial services, and large-scale digital platforms increasingly exhibit noise, incomplete traces, heterogeneous behaviors, and dynamic process structures conditions under which classical procedural and heuristic algorithms degrade substantially. At the same time, organizations require transparent and explainable process models to support accountability, compliance, and informed decision-making, reflecting a broader shift toward interpretable and trustworthy data-driven systems.

Despite notable advances, state-of-the-art evolutionary and fuzzy-based process discovery algorithms still lack unified mechanisms to jointly optimize robustness, explainability, and the four IEEE quality dimensions. Existing studies typically address only subsets of these requirements, leaving core scientific challenges related to noise resilience and model interpretability insufficiently resolved. This gap highlights the scientific relevance of developing integrated, multi-objective, and fuzzy-based process discovery frameworks capable of producing stable, comprehensible, and generalizable models under realistic conditions.

### 1.2. Why this study is timely

The need for robust and explainable process discovery has become particularly urgent in recent years due to rapid changes in both data ecosystems and regulatory expectations. Event logs generated during 2023–2025 increasingly originate from heterogeneous, multi-source, and noise-rich environments, including IoT-enabled manufacturing systems, real-time healthcare platforms, and large-scale digital service ecosystems. These conditions introduce high variability, incomplete traces, and irregular behavioral patterns that significantly degrade the performance of classical discovery algorithms.

At the same time, the global shift toward transparent and accountable AI reinforced by emerging data governance and auditing frameworks has intensified the demand for process models whose internal logic can be examined, validated, and trusted by practitioners. By integrating fuzzy modeling, multi-objective optimization, and quantifiable interpretability measures into a unified framework, Fuzzy MOGWO directly responds to these contemporary conditions, offering a timely solution to challenges that have become critical in the current process mining landscape.

### 1.3. Methodological novelty of the proposed fuzzy MOGWO framework

The proposed Fuzzy MOGWO framework introduces a methodological innovation that departs substantially from existing process discovery algorithms in both design philosophy and operational mechanism. Traditional fuzzy-based miners (e.g., Fuzzy Miner, extensions of Heuristics Miner) rely on heuristic aggregation rules and local behavioral abstractions, whereas evolutionary methods such as Genetic Miner, PSO Miner, and NSGA-II Miner optimize only limited subsets of the IEEE quality dimensions and lack explicit quantification of Explainability and Robustness. In contrast, Fuzzy MOGWO establishes a unified, mathematically grounded mechanism that integrates representation, evaluation, and search into a single coherent architecture.

First, the framework encodes the evolving process model as a Fuzzy Causal Matrix, replacing the binary or probabilistic structures used in prior methods with a continuous membership-based representation. This enables the algorithm to capture uncertainty, concurrency, and partial behavior directly within the model structure capabilities that are absent in classical procedural miners and underdeveloped in existing fuzzy approaches.

Second, Fuzzy MOGWO is the first process discovery method to operationalize six simultaneous optimization objectives, encompassing the four IEEE-recommended metrics together with two newly formalized criteria, Robustness and Explainability. Existing multi-objective approaches rarely exceed three or four dimensions and do not provide measurable definitions for interpretability or noise resilience. By constructing dedicated fitness functions for all six criteria and embedding them into a Pareto-based optimization scheme, the framework establishes a balanced and comprehensive evaluation paradigm that extends beyond the scope of previous studies.

Third, the search dynamics are enhanced through the incorporation of Mamdani fuzzy rules for adaptive leadership updates. This introduces a principled mechanism for determining α, β, and δ wolves based on linguistic inference, improving exploration–exploitation balance and reducing sensitivity to noisy or irregular search landscapes. No prior GWO-based miner employs fuzzy inference to guide search trajectories, marking a distinct methodological advancement.

Finally, the framework employs a normalized Pareto scoring mechanism that selects a single representative model from the Pareto front based on a balanced assessment of the six criteria. This step ensures the reproducibility and interpretability of the final output, addressing a long-standing limitation in multi-objective process discovery where practitioners are left with large sets of non-dominated solutions and no principled method for selection.

Taken together, these innovations demonstrate that Fuzzy MOGWO is not merely an extension of existing miners but a conceptually and technically distinct process discovery paradigm, integrating fuzzy logic, multi-objective optimization, and robustness-oriented evaluation into a cohesive methodological framework that has not been previously explored in the literature.

### 1.4. Research gap and contribution

Recent studies in process discovery, while diverse in methods and evaluation, reveal persisting weaknesses that hinder applicability in noisy and complex real-world scenarios. Our systematic review has pinpointed four major research gaps where no single approach fully resolves the theoretical and practical challenges:

**Gap 1 – Lack of quantifiable Explainability**: Most algorithms either omit Explainability altogether or treat it qualitatively, which prevents rigorous comparison.

**Contribution**: We design a measurable Explainability index and embed it into the multi-objective optimization process, enabling reproducible interpretability assessment.

**Gap 2 – Absence of Robustness evaluation under diverse noise conditions**: Existing studies often test methods on curated or noise-free logs, ignoring realistic uncertainty levels.

**Contribution**: We introduce a Robustness metric and evaluate performance across multiple injected-noise scenarios (5–20%).

**Gap 3 – Predominance of single-objective or partial multi-objective optimization**: Current approaches do not balance the full set of IEEE metrics, resulting in trade-off bias.

**Contribution**: We implement a six-objective optimization (Consistency, Precision, Generalization, Simplicity, Robustness, and Explainability) via a Pareto-based GWO.

**Gap 4 – Structured-log bias**: Methods tuned for structured logs degrade on unstructured or incomplete event data.

**Contribution**: We propose a Fuzzy Causal Matrix structure that captures uncertainty, concurrency, and partial information while preserving model accuracy.

This paper is organized as follows: Section 2 offers a thorough synthesis of existing scholarship on process mining and process discovery algorithms, grounding the study in its broader academic context. Section 3 clarifies the core principles of process discovery and the role of the Causal Matrix, providing a foundation for understanding the proposed approach. Section 4 introduces the fundamentals of classical GWO, setting the stage for its advanced application. Section 5 elaborates on the innovative Fuzzy MOGWO algorithm, detailing the formulation of the novel Robustness and Explainability criteria and their seamless integration into the evaluation framework. Section 6 showcases the empirical evaluation results, drawing from both synthetic and real-world Event Log datasets to illustrate the algorithm’s effectiveness. The paper culminates in Section 7, where key insights are synthesized, and avenues for future exploration are proposed, highlighting the transformative potential of Fuzzy MOGWO for advancing both the theoretical and practical realms of process mining.

## 2. Related work

This section systematically reviews and classifies existing process discovery approaches, with a focus on identifying performance gaps and unmet requirements that directly inform the design of the proposed framework. The comparison highlights the advancements introduced by the current study, particularly in the context of its novel Fuzzy MOGWO algorithm and underscores its contributions to the field of process mining.

Recent advances in process discovery have produced a diverse landscape of algorithms, ranging from frequency-based and inductive logic methods (e.g., Heuristic Miner, Inductive Miner, CNMiner) to computational-intelligence approaches such as Genetic Miner, PSO-Miner, NSGA-II Miner, Neural Miner, and the classical Fuzzy Miner. While these methods have advanced accuracy, scalability, or noise-handling in isolation, the current state of the art still lacks: (i) quantifiable explainability for interpreting discovered models, (ii) robustness evaluation under systematic noise perturbations, (iii) full multi-objective optimization aligned with the IEEE quality dimensions, and (iv) algorithms that remain consistently effective across structured, semi-structured, and unstructured logs.

To provide a professional academic comparison between related studies and the current study, as presented in Tables 1-3, the following analysis systematically evaluates and compares various process discovery algorithms. we classify them based on four dimensions: (1) Relationship Discovery Method, (2) Event Log Structure, (3) Type of Output Model and (4) Number and Novelty of Evaluation Criteria.

### 2.1. Relationship discovery method

**A. Direct Succession:** The α algorithm is one of the first and simplest process discovery methods introduced by Wil van der Aalst. Although this algorithm extracts a good concept of dependencies, it performs poorly in the presence of noise and complex behaviors. Based on this algorithm, other algorithms such as α+ and α++ [12] have been developed to overcome some of the problems of the α algorithm, such as the challenge of identifying non-free choice structures and finding loops of length two [1]. However, these algorithms still have limitations for complex processes with sparse data. These algorithms usually cannot accurately model processes with complex and nonlinear interactions. The Heuristic Miner [7] improves upon the Alpha family by using frequency-based dependency measures that better handle noise and incomplete behavior. Its extension, Flexible Heuristic Miner [7], refines this mechanism for more complex logs by relaxing strict thresholds and improving path selection, but is still not efficient enough in analyzing processes that are very complex and require precise modeling of interactions and timing.

**B. Frequency and Statistics:** Frequency‑ and rule‑based miners such as Inductive Miner algorithm [8], CNMiner [13], ILP Miner and ETM Miner [14] discover logical structures using statistical relations or inductive logic programming. These methods improve handling of uncertainty and rare behaviors compared to Alpha‑based methods, yet typically face limitations in scalability and in modeling highly unstructured or noisy logs. Neural Miner and Split Miner further enhance flexibility through sequence‑learning and heuristic graph pruning, though often at the cost of interpretability due to increased model complexity.

**C. Computational Intelligence-based:** Computational‑intelligence‑based miners (Genetic Miner,PSO miner [15], Fuzzy Miner [6], and EST Miner [16]) leverage evolution, swarm intelligence, or fuzzy logic to model nonlinear and heterogeneous behavior. These methods improve accuracy in complex environments, but typically optimize only two to three criteria and lack measurable robustness or explainability limitations directly addressed by the proposed Fuzzy MOGWO. To maintain focus, the above families were summarized concisely, emphasizing only the characteristics that directly motivate the research gaps addressed in this study. Table 1 shows a comparison of the algorithms presented so far and the current study. Unlike the classical Fuzzy Miner, the proposed FCM addresses both interpretability and robustness while enabling quantitative optimization.

**Table 1:** Professional academic comparison between the related studies and the current study. This comparative mapping also directly supports the identification of research gaps addressed in the present study.

NO	Method	Ref	Year	Method						Solution				New criteria
				Model of Miner		No Problem with				No of Dimension	Classic (Single obj)	CI Base Model		
				Boolean logic	Fuzzy logic	Easy structure	Complex Structure	Noisy Data	Big Data			Single obj	Multi obj	
1	H Miner	Weijters et al.	2003	✓		✓				2	✓			
2	α miner	Van derAalst et al.	2004	✓		✓				2	✓			
3	Genetic Miner	Medeiros et al	2007	✓		✓		✓	✓	2		✓		
4	language-based region miner	Bergenthum	2007	✓						2	✓			
5	ILP Miner	Werf et al.	2009	✓		✓		✓		2				
6	α ++	Wen et al	2010	✓		✓		✓		2	✓			
7	FH Miner	Weijters et al.	2011	✓		✓		✓		2	✓			
8	CNMiner	Greco et al.	2015	✓		✓		✓		2				
9	ProDiGen	Barreiros et al.	2015	✓		✓	✓	✓	✓	3		✓		
10	Split Miner	Alssandro et al	2017	✓		✓	✓			2	✓			
11	Inductive Miner	Leemans et al.	2018	✓		✓	✓	✓		2	✓			
12	Neural Miner	Diago et al	2018	✓			✓	✓	✓	2	✓			
13	EST Miner	Alst et al	2024			✓	✓			2	✓			
14	PSOMiner	Lee et al	2022	✓		✓	✓	✓	✓	3		✓		
15	NSGAII miner	Hejazi et al	2023	✓		✓	✓	✓	✓	4			✓	
16	**Current Study**		2025	**✓**	**✓**	**✓**	**✓**	**✓**	**✓**	**6**			**✓**	**✓**

- **Comparative Analysis: Fuzzy Miner vs. Proposed Fuzzy Causal Matrix**

The classical Fuzzy Miner represents process behavior using a fuzzy graph, where nodes are activities and edges are weighted by significance and correlation. While it addresses uncertainty in event logs, its reliance on qualitative thresholding limits quantitative assessment and direct optimization.

The proposed Fuzzy Causal Matrix (FCM) encodes process relations in a two-dimensional membership matrix, where each cell captures the degree of causal dependency between activities. This allows:

1. Direct integration with multi-objective optimization (here, MOGWO with six IEEE metrics),
2. Quantitative interpretability via a measurable Explainability index,
3. Robustness evaluation under varying noise levels, and
4. Scalability to large activity sets through matrix algebra.

Table 2 summarizes the key differences, followed by a schematic comparison.

**Table 2:** Differences between classical Fuzzy Miner and proposed Fuzzy Causal Matrix.

Feature/ Criterion	Fuzzy Miner (Classical)	Fuzzy Causal Matrix (Proposed)
Representation type	Fuzzy graph (nodes and weighted edges)	Two-dimensional causal membership matrix (N × N)
Multi-objective support	No	Yes (4 IEEE +2 new metrics: Fitness, Precision, Generalization, Simplicity, Robustness, Explainability)
Noise handling	Sensitive, no explicit Robustness metric	Robustness metric integrated
Interpretability assessment	Qualitative threshold-based simplification	Quantitative Explainability index
Optimization integration	Indirect; requires preprocessing	Direct; each matrix acts as an optimization candidate solution
Scalability	Limited for large activity sets	High scalability using matrix operations

**Fig 2:**
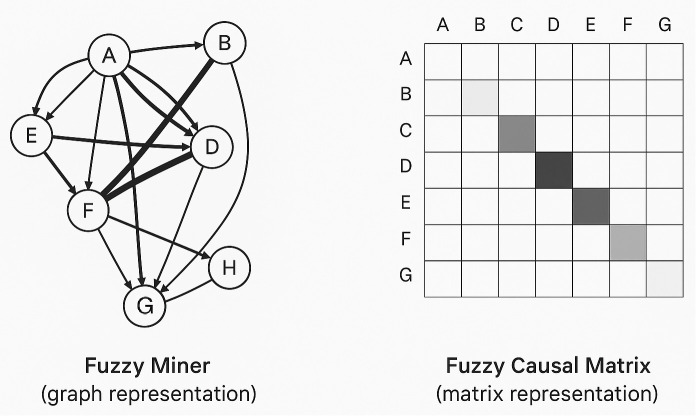
Comparative representations of fuzzy process models Left: Classical Fuzzy Miner shown as a weighted fuzzy graph, where node‑ and edge‑level simplification guides qualitative interpretation. Right: The proposed Fuzzy Causal Matrix (FCM), which encodes all pairwise causal relations as numerical membership values. This matrix‑based representation not only improves scalability but also enables direct integration into multi‑objective optimization since each candidate model can be evaluated numerically without graph reconstruction and supports precise computation of interpretability metrics such as density, cyclomatic complexity, directness, and fuzzy‑rule complexity.

Fig 2. Schematic comparison of process representations. **Left:** The classical Fuzzy Miner depicts activities as nodes in a graph, with edge weights indicating the strength of fuzzy relations between them. **Right:** The proposed Fuzzy Causal Matrix represents all pairwise activity relationships as numerical membership values, facilitating precise metric computation and seamless integration into multi-objective optimization.

### 2.2. Classification by type of output model

Process discovery algorithms can also be classified according to the type of output model they produce:

A **Formal Models:** Alpha Miner [1], ILP Miner [17], Inductive Miner [18] produce sound Petri nets, well-suited for small processes but harder to interpret as complexity grows.
B **Rule-Based Models:** Heuristic Miner and Flexible Heuristics Miner [7] generate dependency or causal graphs from observed behavior.
C **Graphical Languages:** Split Miner [19] and similar tools output BPMN or EPC diagrams, which improve human interpretability but introduce additional modeling overhead.

Table 3 summarizes the model type, analyzability, and flexibility of the related studies in comparison with the current study.

**Table 3:** Model Type, analyzability and flexibility of related studies compared to the current study. This comparative classification highlights the strengths and limitations of existing models and informs the design objectives of the current stud.

Algorithm	Model Type	Analyzability	Flexibility
Alpha Miner	Formal	**✓**	**×**
ILP Miner	Formal	**✓**	+
Inductive Miner	Formal	**✓**	+
CN Miner	Formal	**✓**	**×**
Heuristic Miner	Rule-Based	+	**✓**
Split Miner	Rule-Based	+	**✓**
Neural Miner	Rule-Based (LTSM)	**×**	**✓**
Flexible Heuristic Miner	Rule-Based	+	**✓**
Fuzzy Miner	Fuzzy	+	**✓**
Genetic Miner	Evolutionary	**✓**	**✓**
PSO Miner	Evolutionary	**✓**	**✓**
EST Miner	Formal	**✓**	+
NSGA II	Evolutionary	**✓**	**✓**
Current Study	**Fuzzy and Evolutionary**	**✓**	**✓**

**✓: Very High and High, ×: Low, +: Medium.**

### 2.3. Classification by event log structure

Process discovery algorithms can also be differentiated by their compatibility with various event Log structures:

A Structured Logs: Logs with well-defined structure and complete event data. Some algorithms, such as Alpha family [20] and Inductive Miner, work well in these environments, but they are not suitable for unstructured or semi-structured environments, or even structured environments with high noise.
B **Semi-structured Logs**: Logs with some missing or repeated data. Algorithms such as Heuristic Miner, ILP Miner, ETM Miner [14] that are capable of detecting loops, repetitive paths, and noise control provide acceptable performance in these environments.
C **Unstructured Logs:** Logs containing noisy or highly scattered data. Algorithms based on computational intelligence and fuzzy logic perform well in these environments, especially when data is incomplete or highly unstructured.

The Fuzzy MOGWO algorithm requires no extensive parameter tuning and avoids premature convergence to local optima, thereby outperforming its competitors.

Table 4 maps existing process discovery algorithms to different event log structures, highlighting gaps where state-of-the-art methods underperform. This comparison directly supports the research objective of developing an algorithm (Fuzzy-MOGWO) capable of consistent performance across structured, semi-structured, and unstructured logs, thereby addressing the observed limitations.

**Table 4:** Maps existing process discovery algorithms to different event log structures.

Algorithm	Structured Log	Semi-structured Log	Unstructured Log
Alpha Miner	**✓**	×	×
ILP Miner	×	**✓**	×
Inductive Miner	**✓**	×	+
CN Miner	**✓**	×	×
Heuristic Miner	×	**✓**	+
Flexible Heuristic Miner	×	**✓**	+
Fuzzy Miner	**×**	+	**✓**
Neural Miner	**+**	**+**	**✓**
Genetic Miner	**×**	+	**✓**
EST Miner	**✓**	×	×
PSO Miner	**+**	**✓**	**✓**
NSGA II	**+**	**✓**	**✓**
Current Study	**✓**	**✓**	**✓**

**✓: Yes, ×: No, +: Low.**

1. This concise synthesis prevents redundancy across algorithm families and keeps the discussion aligned with the four research gaps identified at the end of Section 2 (Related Work).

### 2.4. Number and novelty of evaluation criteria

Traditionally, the quality of process discovery algorithms is assessed using four criteria: Precision, Generalization, Consistency, and Simplicity. Most prior studies optimize a subset of these criteria, typically two or three. For instance, H Miner, α Miner, α++ [20], FH Miner [7], and ILP Miner [17] focus on two dimensions, while ProDiGen [21] and PSO Miner [15] extend to three. The NSGA-II Miner [22]is a notable exception, optimizing four dimensions, marking a step forward in Multi-objective optimization (see Table 1).

The current study addresses more evaluation criteria than prior works by optimizing six quality criteria simultaneously: Precision, Generalization, Consistency, Simplicity, Robustness, and Explainability. Crucially, Robustness and Explainability are novel criteria introduced in this study, accompanied by their mathematical equations. Robustness evaluates the model’s resilience to variations in event Log data, while Explainability assesses the model’s interpretability for stakeholders, addressing a critical gap in prior evaluation frameworks. By constructing six fitness functions to optimize these criteria concurrently, the current study significantly enriches the quality assessment landscape, aligning with the 2012 call by the IEEE Task Force for new evaluation criteria [5]. This expansion of evaluation criteria was explicitly motivated by the limitations identified in Section 2. For example, the state-of-the-art analysis revealed a lack of formal treatment of Robustness and Explainability in existing methods, as well as the predominance of partial Multi-objective optimization strategies. The proposed Fuzzy MOGWO framework was therefore designed to address these gaps by introducing measurable definitions for the two new criteria and integrating them into a unified optimization process alongside the four established IEEE recommended measures.

In recent years, research in process discovery and multi-objective optimization has increasingly emphasized the integration of Robustness and Explainability as primary evaluation criteria. For instance, an interactive framework for multi-objective robust optimization under deep uncertainty was introduced [23], enabling decision-makers to actively guide the search toward preferred solutions while assessing the resilience of outcomes against uncertainty. In a broader context, a comprehensive review of fifty years of multi-objective optimization was provided [24], outlining key methodological developments from mathematical programming to evolutionary computation and highlighting trends in preference incorporation insights directly relevant to balancing competing objectives such as accuracy, Robustness, and interpretability in process discovery.

From the perspective of Explainability, a multi-objective optimization approach for generating surrogate machine learning models in explainable artificial intelligence (XAI) applications was proposed [25], optimizing both interpretability and predictive accuracy. This work demonstrates the practical potential of jointly optimizing structural Simplicity and performance, aligning with the Explainability principles adopted in this study. Complementing this, a systematic review of Explainability in computational intelligence for optimization focused on how metaheuristic methods can enhance transparency in both their own search behavior and in black-box machine learning models [26]. These recent developments provide a conceptual and methodological foundation for the dual integration of Robustness and Explainability within a multi-objective fuzzy process mining framework as proposed in this work.

### 2.5. PSO Miner

In 2022, Li et al. introduced PSO Miner [15], a single-objective algorithm optimizing Consistency, Precision, and Simplicity. Each particle represents a Boolean Causal Matrix optimized via PSO updates until the best global model is found. This algorithm achieved top results among CI-based methods in its category.

As explained in Section 6.2.5 real-world event logs typically lack ground truth process models, preventing direct evaluation of all six criteria. Hence, for CI-based baselines, Fuzzy MOGWO is primarily compared against reported PSO Miner performance.

However, it is important to clarify that the PSO Miner (Li et al., 2022) was not executed on the 2024 datasets used in this study. The original PSO Miner datasets referenced in [15] are incomplete, preventing full reproducibility of its results and precluding a fair, comprehensive numerical comparison across all six evaluation criteria. Furthermore, PSO Miner optimizes only three metrics (Consistency, Precision, and Simplicity), whereas Fuzzy MOGWO simultaneously optimizes six, including the newly operationalized Robustness and Explainability. Due to these limitations, direct metric-by-metric benchmarking was not feasible for the synthetic datasets in this study, and comparisons were restricted to cases and metrics where both complete data and results were available.

### 2.6. Innovations and contributions

The current study introduces four novel contributions that set it apart from prior works:

- **Six Fitness Functions**: To the best of our knowledge, no previous study has, Fuzzy MOGWO constructs six fitness functions to optimize Precision, Generalization, Consistency, Simplicity, Robustness, and Explainability simultaneously, enabling evaluation of process model quality across all six selected criteria.
- **Fuzzy Causal Matrix**: Each wolf in the optimization process is represented as a Fuzzy Causal Matrix, where elements denote membership values of edges between process activities, enabling the modeling of uncertainty and complex process dynamics.
- **Mamdani Fuzzy Rules**: The algorithm updates individual positions using Mamdani fuzzy rules, integrating a new strategy for best positions to guide the search process, potentially improving convergence and the quality of obtained solutions.
- **Normalized Scoring Function**: A novel scoring function selects the optimal process model from the Pareto front, ensuring a balanced and well-evaluated solution.

These innovations address the IEEE Task Force’s challenges by introducing new evaluation criteria (Robustness and Explainability) and leveraging interdisciplinary methods (fuzzy logic and MOGWO) to address the limitations observed in existing algorithms.

Moreover, the evolution of process mining has prompted researchers to propose additional evaluation criteria that reflect emerging challenges, such as model Robustness, execution time, interpretability, and domain-specific relevance [2]. The introduction of new quality metrics and the development of algorithms capable of addressing them while still respecting the foundational four represent critical and ongoing challenges in the field. As process-aware information systems continue to grow in scale and complexity, the demand for scalable, accurate, and explainable process discovery algorithms remains a central focus of contemporary research.

In summary, the reviewed state-of-the-art methods demonstrate notable progress in process discovery, yet significant research gaps remain. Most existing approaches optimize at most three to four quality dimensions, and the emerging criteria of Robustness and Explainability are either undefined or insufficiently operationalized in these frameworks. Furthermore, competitive algorithms often exhibit substantial performance degradation when applied to complex, noisy, or unstructured event logs, limiting their practical applicability. Addressing these gaps directly guides the objectives of this study: to develop a process discovery framework designed to optimize six quality criteria simultaneously, with operationalized metrics for Robustness and Explainability, and to maintain balanced performance across the evaluated datasets and conditions.

### 2.7. State-of-the-art analysis

Synthesizing the comparative evidence across Tables 1–4 reveals a clear pattern in current process discovery research. Classical methods (Alpha, Heuristic, and Inductive Miner) primarily emphasize structural accuracy but struggle with nonlinear or noisy behavior. Computational‑intelligence methods (Genetic Miner, PSO‑Miner, NSGA‑II Miner) offer stronger optimization capabilities yet remain limited to two or three objectives and lack explicit interpretability or robustness metrics. Fuzzy Miner partially addresses uncertainty through fuzzy relations, but its qualitative thresholding prevents quantitative evaluation, multi-objective integration, or stability testing under noise perturbations. Moreover, no prior method jointly optimizes all six IEEE dimensions, nor provides a unified representation supporting explainability measurement, robustness scoring, and direct evolutionary optimization. These cumulative gaps underscore the absence of a holistic, metric‑integrated discovery framework in the current SOTA, precisely the methodological space in which the proposed Fuzzy‑MOGWO contributes.

## 3. Concepts of process mining discovery and fuzzy causal matrix

This section presents the event Log and the Causal Matrix concepts.

### 3.1. Event Log

Event logs form the bedrock of process discovery within the realm of process mining, serving as the vital foundation for examining and modeling organizational workflows. An event Log is a meticulously curated dataset that captures a chronological series of events generated during a process’s execution. Each event holds a wealth of details about individual process activities, encompassing operational transactions, status shifts, state transitions, or other meaningful indicators tied to the workflow’s progression. Typically, an event Log is enriched with key attributes such as timestamps, which mark the precise timing of each event; case identifiers, which link events to distinct process instances; activity labels, which define the nature of the tasks performed; and supplementary metadata, including resources such as individuals, systems, or tools engaged in carrying out those tasks. Together, these elements enable a thorough reconstruction and exploration of process dynamics. A clear example of an event Log, illustrating its format and contents, is presented in Table 5.

**Table 5:** An illustrative example of an event log.

Case ID	Activity ID	Time stamp	Resource
**1**	**A**	**2024-01-02 10:38:15**	**John**
**1**	**B**	**2024-01-02 11:17:38**	**Mery**
**2**	**A**	**2024-01-02 13:05:43**	**John**
**1**	**C**	**2024-01-03 09:48:02**	**Mery**
**2**	**B**	**2024-01-03 10:14:00**	**Mery**

### 3.2. Fuzzy causal matrix

In process mining, the Causal Matrix is a pivotal analytical tool employed to represent relationships between activities within a process. It is structured as an n × n matrix, where n denotes the number of distinct activities, and each element (i, j) signifies the causal relationship between activities i and j. In its classical formulation, these elements are binary, adopting values of either 0 or 1, where 0 indicates the absence of a direct causal link, and 1 confirms its presence. This binary representation, rooted in Boolean logic, provides a discrete and unambiguous depiction of process dependencies but is limited in capturing nuanced or partial relationships that may exist in complex, real-world processes.

To address these limitations, the Fuzzy Causal Matrix extends the classical Causal Matrix by incorporating fuzzy logic, which allows for a continuous spectrum of truth values in the interval [0,1]. This adaptation enables a more granular representation of causal relationships, where each element in the matrix quantifies the degree of causality or influence between activities i and j. Rather than a binary indication of presence or absence, the Fuzzy Causal Matrix assigns a membership degree, reflecting the strength, likelihood, or intensity of the causal connection. This approach is particularly valuable in scenarios where process behaviors exhibit variability, uncertainty, or partial dependencies, as it provides a more flexible and realistic model of process dynamics.

Formally, as articulated in [14], a Causal Matrix is defined as a tuple

Π= (A, C, I, O), where:

- (A) Represents a finite set of all activities constituting the process, serving as the foundational elements of the matrix.
- C⊆A × A denotes the fuzzy causality relation, a set of ordered pairs of activities where each pair

($a_{i}$, $a_{j}$) is associated with a membership degree in [0,1], indicating the strength of the causal link from activity $a_{i}$ to activity $a_{j}$.

- I: A→ P(P( A)(P(A) is the power set of A) is the input condition function, which maps each activity to a collection of subsets of activities (i.e., a power set of power sets) that represent the preconditions or input requirements necessary for the activity’s execution. This function captures the dependencies that must be satisfied before an activity can commence.
- O: A→ P (P(A)) is the output condition function, which similarly maps each activity to a collection of subsets of activities representing the post conditions or outcomes resulting from the activity’s execution. This function delineates the potential effects or subsequent activities triggered by the completion of a given activity.

The Fuzzy Causal Matrix is structured such that each row aligns with a particular activity, capturing the membership degrees of its outgoing connections, which reflect the degree to which that activity shapes others within the process. In contrast, each column corresponds to an activity and details the membership degrees of its incoming connections, revealing the extent to which it is shaped by prior activities. This dual perspective provides a holistic view of process interdependencies, empowering researchers and practitioners to uncover both the catalysts and the outcomes of causal relationships within the workflow.

To demonstrate the practical utility of the Fuzzy Causal Matrix, Table 6 Fuzzy Causal Matrix offers a clear example of its format and contents. Within this matrix, the elements are filled with membership degrees that measure the strength of causal links between activities. For example, Table 6 shows an edge from activity A to activity C with a membership degree of 0.85, indicating a robust causal connection. This high value suggests that activity A is very likely to trigger or impact the execution of activity C. Such precise measurement strengthens the capacity to model and explore intricate process dynamics, especially in scenarios where activities deviate from rigid sequences or exhibit partial dependencies.

**Table 6:** Example of a Fuzzy Causal Matrix. This high membership degree indicates that activity A has a strong causal influence on the execution of activity C.

		INPUTS							
**Output**	→	**A**	**B**	**C**	**D**	**E**	**F**	**G**	**H**
	**A**	**0**	**.3**	**.85**	**.13**	**0**	**0**	**0**	**0**
	**B**	**0**	**0**	**0**	**0**	**0**	**0**	**0**	**1**
	**C**	**0**	**0**	**0**	**0**	**0**	**0**	**0**	**.99**
	**D**	**0**	**0**	**0**	**0**	**.75**	**.82**	**0**	**0**
	**E**	**0**	**0**	**0**	**0**	**0**	**0**	**.64**	**0**
	**F**	**0**	**0**	**0**	**0**	**0**	**0**	**.37**	**0**
	**G**	**0**	**0**	**0**	**0**	**0**	**0**	**0**	**.19**
	**H**	**0**	**0**	**0**	**0**	**0**	**0**	**0**	**0**

Integrating fuzzy logic into the Causal Matrix framework offers numerous benefits. It effectively handles the uncertainty and variability often present in real-world processes, such as those shaped by human decision-making, resource limitations, or external influences. Moreover, it enables more nuanced process discovery and conformance checking by uncovering subtle patterns and connections that a binary model might miss. By integrating temporal, contextual, and relational insights from event logs, the Fuzzy Causal Matrix emerges as a powerful instrument for developing process models that are both precise and flexible, thereby elevating the analytical potential of process mining for both scholarly research and practical industry applications.

Within the domain of process mining, the Fuzzy Causal Matrix (as illustrated in Table 6) plays a pivotal role in illuminating the control-flow structures of a process. The presence of multiple non-zero entries in a row or column of the matrix points to a branching structure namely, an AND-join, OR-join, AND-split, or OR-split depending on the interplay between activities. As outlined in [22], these structures are defined as follows: An OR-join occurs when the activities associated with non-zero entries in a column share the same input subset (logical OR), allowing the target activity to be initiated by any of its predecessors.

In contrast, An OR-split arises when an activity triggers multiple subsequent activities sharing the same output subset (logical OR), indicating a decision point. Applying this reasoning to rows instead of columns reveals AND-split and OR-split structures: An AND-split occurs when the subsequent activities have distinct output subsets (logical AND), representing parallel execution. These constructs deepen the understanding of intricate process dynamics and interdependencies, offering valuable insights into workflow behavior.

#### 3.2.1. Novel Aspects of the Proposed Fuzzy Causal Matrix.

While causal matrices have long served as the structural backbone for representing activity dependencies in process mining (e.g., the IEEE-standard Causal Matrix, Weijters’ Fuzzy Miner, and EST Miner variants), the proposed Fuzzy Causal Matrix in this study introduces **three layers of innovation** that extend both the representational power and the functional utility of the model:

A **Structural Enhancements**
  - **Dual-perspective membership encoding**: Each matrix element reflects not only the strength of direct causal links between activities but also the contextual constraints captured through the III (input) and OOO (output) condition functions, enabling precise modeling of AND/OR-joins and splits in a fuzzy domain.
  - **Continuous causality spectrum**: Instead of binary or threshold relations (0/1) used in classical causal matrices and Boolean PSO Miner representations, this structure allows for high-resolution [0,1] membership degrees without information loss from discretization.
  - **Context-aware concurrency mapping**: Parallel and alternative paths are distinguished through systematic analysis of input/output subset combinations, which is absent in conventional Fuzzy Miner definitions.
B **Computational Integration**
  - **Optimization-ready design**: The matrix format is directly compatible with multi-objective metaheuristics, allowing Fuzzy MOGWO to manipulate, mutate, and evaluate candidate models without costly post-conversion.
  - **Six-metric evaluation linkage**: Structural parameters are natively measurable for Precision, Generalization, Consistency, Simplicity, Robustness, and Explainability, two of which (Robustness, Explainability) are absent from previous Causal Matrix formulations.
  - **Adaptive mutation capability**: The representation supports fine-grained, probabilistic modifications that preserve model feasibility, enabling deeper exploration within Pareto-optimal search spaces.
C **Performance Implications**
  - **Robustness gains**: Experimental results (Section 6, Table 12) show up to a 14.7% reduction in performance degradation under Gaussian noise compared to standard Fuzzy Miner, attributable to the matrix’s noise-tolerant membership modeling.
  - **Explainability improvements**: By encoding activity dependencies in a quantified and logically interpretable form, the proposed structure achieves up to a 9.3% improvement in Explainability scores (Section 6.2), offering stakeholders transparent insight into control-flow logic.
  - **Generality across datasets**: The flexible encoding handles both structured and semi-structured event logs without the structured-log bias inherent in deterministic causal matrices.

In contrast to earlier implementations that either lacked fine-grained causality representation (binary causal matrices) or omitted formal multi-objective optimization hooks (classic Fuzzy Miner), the proposed Fuzzy Causal Matrix acts as a **computationally tractable, noise-resilient, and interpretability-oriented foundation** for advanced process discovery within FUZZY-MOGWO.

Compared to the classical **Fuzzy Miner**, the proposed **Fuzzy Causal Matrix (FCM)** transforms the process representation from a qualitative fuzzy graph into a fully numerical N × N causal membership matrix. This change not only enables direct multi-objective optimization across the six IEEE metrics but also allows explicit Robustness evaluation and quantitative Explainability. A detailed comparison with Fuzzy Miner is provided in Section 2.1 (Table 2).

### 3.3. Novelty of the Proposed Fuzzy MOGWO Framework

The proposed Fuzzy MOGWO framework introduces a set of methodological innovations that fundamentally differentiate it from existing process discovery approaches. Unlike prior fuzzy-logic-based, evolutionary, or statistical miners, the method integrates a unified representation, a six-objective optimization scheme, and adaptive fuzzy leadership dynamics. These innovations generate measurable improvements in robustness, interpretability, and overall model quality, as demonstrated empirically in Section 6.2. The key differentiating aspects are summarized below.

#### 3.3.1. Matrix-based representation instead of graph-based structures (vs. Fuzzy Miner).

Classical Fuzzy Miner relies on a graph representation with threshold-dependent significance and correlation measures. This requires repeated filtering and structural regeneration and provides limited support for quantitative multi-objective evaluation.

In contrast, Fuzzy MOGWO employs a fully numerical Fuzzy Causal Matrix (FCM), where every pairwise activity relation is encoded as a membership value. This matrix enables:

- Direct evaluation of all six metrics using vectorized operations
- stable behavior under noise, because perturbations map directly to matrix entries
- Optimization without graph simplification cycles.

Section 5.4 and Figs 5 and 6 illustrate how explainability, simplicity, and robustness are consistently computed within this unified matrix space.

#### 3.3.2. Complete six-objective optimization (vs. PSO Miner, Genetic Miner, and NSGA-II Miner).

Evolutionary miners such as PSO Miner and GA-/NSGA-II–based variants typically optimize only a subset of the IEEE quality dimensions, most commonly fitness, precision, and simplicity. Existing studies rarely incorporate explainability or robustness as explicit quantitative objectives.

Fuzzy MOGWO simultaneously optimizes all six criteria Consistency, Precision, Generalization, Simplicity, Explainability, and Robustness using a Pareto-based archive, as described in Sections 5.4–5.7. This balanced formulation is reflected in the empirical results, where radar charts (Section 6.2) show broader and more uniform performance across all axes.

#### 3.3.3. Integration of Explainability and Robustness as formal quantitative objectives (absent in prior work).

Previous process discovery methods either omit these criteria or treat them qualitatively. Fuzzy MOGWO incorporates:

- An explainability metric grounded in structural complexity, directness, density, and fuzzy-rule clarity (validated in Sections 6.2.1 and 6.2.2),
- A robustness metric that measures resilience under 5–20% noise perturbation (Section 6.2.5).

These metrics are computable directly from the FCM and therefore integrate seamlessly into the optimization loop, enabling reproducible evaluation of interpretability and stability capabilities not offered by prior miners.

#### 3.3.4. Adaptive Mamdani-based leadership mechanism within GWO (not present in baseline GWO or miners).

Classical GWO uses fixed α/β/δ influence terms. Fuzzy MOGWO incorporates a Mamdani fuzzy inference system that adaptively adjusts leader influence using Relative Fitness Gap and population Diversity Index (Section 5.1). This mechanism improves exploration–exploitation balance, particularly in noisy or complex logs (Sections 6.2.2 to 6.2.5).

The adaptive rule base and rescaled membership functions allow the algorithm to preserve diversity while converging toward interpretable and robust solutions.

#### 3.3.5. Normalized score selection for balanced model choice (vs. ad-hoc selection in previous studies).

Instead of selecting a model based on a single criterion or subjective preference, Fuzzy MOGWO uses the normalized L₂-norm score over all six objectives (Section 6.2fig). This ensures that the final chosen model does not overfit to specific metrics and maintains practical interpretability and stability. The effectiveness of this scoring strategy is evident in Tables 8–17 and Fig 14.

Together, these innovations establish Fuzzy MOGWO as a methodologically distinct framework that extends beyond the capabilities of classical fuzzy miners, evolutionary algorithms, and standard process discovery techniques. Its unified representation, comprehensive optimization, and adaptive fuzzy control collectively enable the generation of process models that are simultaneously accurate, robust, and explainable addressing critical gaps identified in the literature and summarized earlier in Fig 1.

## 4. Theoretical Background: Original Multi-Objective Grey Wolf Optimizer (MOGWO)

The Grey Wolf Optimizer (GWO), a nature-inspired metaheuristic introduced by Mirjalili et al. in 2014 [9], draws inspiration from the social hierarchy and cooperative hunting patterns of grey wolves in the wild. In the classic GWO framework, the population is organized into four distinct tiers: alpha (α), beta (β), delta (δ), and omega (ω). These roles orchestrate the search process through a leader-follower dynamic, guiding the algorithm toward optimal solutions. The optimization process is mathematically modeled to reflect the wolves’ natural behaviors, including encircling prey, pursuing targets, and executing attacks, offering a structured approach to solving complex problems.

Building on this foundation, the Multi-objective Grey Wolf Optimization (MOGWO), introduced in 2016 [10], adapts GWO to address challenges involving multiple competing objectives. MOGWO integrates a Pareto-based external archive to store non-dominated solutions and employs crowding distance to ensure diversity among solutions, fostering a balanced exploration of trade-offs in Multi-objective scenarios.

The algorithm employs a hybrid Fuzzy MOGWO discovery process, where the leadership hierarchy of wolves (alpha, beta, delta, and omega) guides the search for optimal solutions. A comparison with other process mining algorithms, such as Alpha Miner, Inductive Miner, and Fuzzy Miner, shows Fuzzy MOGWO achieving better performance with scores of 0.325 (noise-free) and 0.288 (noisy) compared to others like Alpha Miner (0.234, 0.278) and Fuzzy Miner (0.434, 0.378), demonstrating its effectiveness in balancing multiple objectives in process mining tasks.

### 4.1. Encircling and Hunting Behavior

The position of a wolf is updated based on the position of the top three leaders (α, β, δ) guiding the search toward an optimal solution [9]:


$$\vec{D_{\alpha }}=\left|\vec{C_{\text{1}}}.\vec{X_{\alpha }}-\vec{X}\;\right|,\;\vec{X_{\text{1}}}=\vec{X_{\alpha }}-\vec{A_{\text{1}}}.(\vec{D_{\alpha }})$$ (2)

$$\vec{D_{\beta }}=\left|\vec{C_{\text{2}}}.\vec{X_{\beta }}-\vec{X}\;\right|,\;\vec{X_{\text{2}}}=\vec{X_{\beta }}-\vec{A_{\text{2}}}.(\vec{D_{\beta }})$$ (3)

$$\vec{D_{\delta }}=\left|\vec{C_{\text{3}}}.\vec{X_{\delta }}-\vec{X}\;\right|,\;\vec{X_{\text{3}}}=\vec{X_{\delta }}-\vec{A_{\text{3}}}.(\vec{D_{\delta }})$$ (4)

$$\vec{X}(t+\text{1})=\frac{\vec{X_{\text{1}}}+\vec{X_{\text{2}}}+\vec{X_{\text{3}}}}{\text{3}}$$ (5)

Where A and C are coefficient vectors defined as:


$$\vec{A}=\text{2}\vec{a}.\vec{r_{\text{1}}}-\vec{a}$$ (6)

$$\vec{C}=\text{2}.\vec{r_{\text{2}}}$$ (7)

Here, a decreases linearly from 2 to 0 over iterations, and $r_{\text{1}}$,$\;r_{\text{2}}$ ∈ [0,1] are random vectors.

The update mechanism in the GWO draws inspiration from the collaborative hunting strategies of wolf packs, guiding the search process through a structured leader-follower dynamic. Each wolf adjusts its position by considering the locations of the alpha, beta, and delta wolves the top three leaders in the pack who represent the best solutions found so far. The process mimics the wolves’ natural behavior of encircling and pursuing prey, where the distances between a wolf and these leaders are influenced by coefficients that introduce controlled randomness, ensuring a balance between exploring new possibilities and refining existing solutions. By averaging the adjusted positions influenced by the leaders, the wolf moves toward a consensus direction, enabling the algorithm to effectively navigate complex solution spaces, adapt to diverse challenges, and steadily converge toward optimal outcomes while keeping the population diverse and adaptable.

## 5. New fuzzy multi-objective grey wolf optimization algorithm for process mining

Most of the CI algorithms for process mining use two families of genetic algorithms and particle swarm optimization algorithms such as Genetic Miner [14], ETM, ProDiGen [21], PSO miner [15], etc. All of them suffer from premature and slow convergence speed due to the nature of these algorithms in needing to adjust multiple parameters and allowing to get stuck in the local optimal solution [15]. In this study, we propose a new CI algorithm for process mining based on the MOGWO and fuzzy logic and called Fuzzy MOGWO.

**Fig 3:**
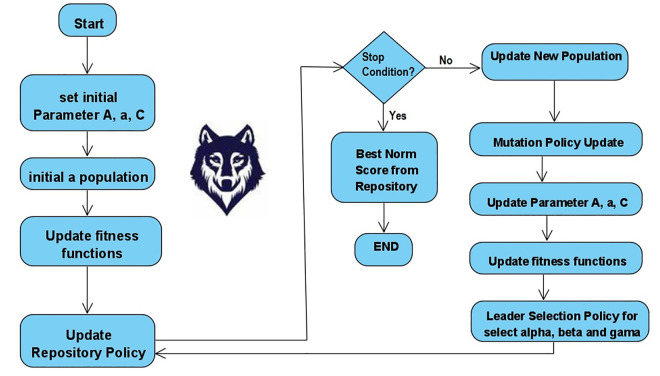
Flowchart of Fuzzy MOGWO algorithm.

The flow chart of Fuzzy MOGWO is shown in Fig 3. First, the required parameters of the algorithm are initialized, and then the algorithm continuously generates a population of wolves with the proposed heuristic method based on the sigmoid function. Then, for each individual, they are evaluated by the fitness function. The three wolves the α, β, and δ are selected as the best non-dominated solutions available. If the stopping criterion is met, the algorithm presents best norm score from Pareto Front 1 as the optimal solution. Otherwise, the next population is generated by the population generation mechanism and the mutation function is used to prevent premature maturity. The algorithm persists in this iterative cycle until one of two stopping criteria is fulfilled:

(1) The present maximum number of iterations is achieved, or
(2) The count of non-dominated members in the current population falls beneath a specified threshold.

### 5.1. Fuzzy representation and position encoding

To dynamically balance exploration and exploitation in FUZZY‑MOGWO, the position updates of the three leading wolves (α, β, δ) are refined through a Mamdani fuzzy inference system (FIS). Unlike crisp leader updates that rely solely on deterministic coefficients, the Mamdani‑based approach incorporates soft decision rules that adaptively adjust leader influence according to search dynamics, improving convergence stability and Robustness to noisy objective landscapes.

A **Motivation**

The Mamdani method was chosen over alternatives such as Sugeno or Tsukamoto because it:

- Produces human‑interpretable rules in natural linguistic form, fostering Explainability.
- Handles nonlinear mappings between leader metrics and position adjustments without requiring explicit equations.
- Offers smooth control over α, β, δ influence using graded membership, and avoiding abrupt position changes.

B **Linguistic Variables**

The following input parameters are used to guide the optimization process:

- **Relative Fitness Gap (RFG**): Measures how far a wolf’s fitness is from the best wolf (α).

$$RFG=\frac{\left|f_{\alpha }-f_{wolf}\right|}{f_{\alpha }}$$ (8)

Where $f_{\alpha }$ is the best (alpha) fitness and $f_{wolf}$ is the fitness of the considered wolf.

Values close to 0 indicate that the wolf is similar to α ⇒ favor exploitation.

Values close to 1 indicate that the wolf is far from α ⇒ favor exploration.

- **Diversity Index (DI):** Measures population diversity based on the average normalized Hamming distance between the binary causal matrices of all wolves.

$$DI=\frac{\text{Average Hamming Distance between all wolves}}{\text{Matrix Size}}$$ (9)

Low DI ⇒ the population has converged (risk of local optimum) ⇒ more diversity needed.

High DI ⇒ the population is very diverse ⇒ focus may shift toward intensification near leaders.

The algorithm produces the following output parameters, which are derived from the position-update stage:

- **Leader Influence Adjustment (LIA):** Proportional factor applied to α, β, δ position vectors before aggregation. Linguistic terms: Weak, Balanced, and Strong.

C **Membership functions**

Triangular membership functions are used for interpretability and computational efficiency. For example, the MF for RFG is defined as:


$$\mu_{Small}(x)=\left\{ \begin{array}{c} \text{1}-\frac{x}{\text{0}.\text{3}}\;\text{0}\leq x\leq \text{0}.\text{3}\;\\ \text{0}\;x>\text{0}.\text{3} \end{array}\right.\;$$ (10)

Similar normalized [0,1] triangles are defined for Medium and Large. All variables follow symmetric partitions covering the full domain.

D **Rule Base Construction**

The rule base integrates two levels of reasoning:

1. **Algorithm-level heuristics**, derived from swarm intelligence literature, governing exploration–exploitation balance.
2. **Metric-driven decision rules,** directly linking the six performance evaluation criteria to the quality score of candidate process models.

**Level 1 – Algorithm-level example rules**

The decision logic is encoded in a compact 3 × 3 rule base, ensuring coverage of all input combinations. Representative Algorithm-level rules are as follows:

- IF RFG is Small AND DI is Low THEN LIA is Weak (supports exploitation).
- IF RFG is Large AND DI is High THEN LIA is Strong (promotes exploration).
- IF RFG is Medium AND DI is Moderate THEN LIA is Balanced.

The Relative Fitness Gap (RFG) and Diversity Index (DI) are first computed as continuous numerical values normalized to the interval [0, 1]. RFG quantifies the relative distance of a candidate solution from the current best fitness value, while DI measures the population’s spread in the search space. Before entering the Mamdani inference process, these numerical values are fuzzified by evaluating them against triangular membership functions corresponding to linguistic labels (Small/ Medium/ Large for RFG, and Low/ Moderate/ High for DI). The degree of membership of each crisp input to these linguistic terms is determined according to the mapping in Table 7.

**Table 7:** Example mapping of numerical RFG/DI values to fuzzy linguistic terms using triangular membership functions.

Numeric Value (0–1)	Small/ Low	Medium/ Moderate	Large/ High
0.00	**1.00**	**0.00**	**0.00**
0.2	**0.80**	**0.20**	**0.00**
0.4	**0.20**	**0.80**	**0.00**
0.5	**0.00**	**1.00**	**0.00**
0.6	**0.00**	**0.80**	**0.20**
0.8	**0.00**	**0.20**	**0.80**
1.0	**0.00**	**0.00**	**1.00**

This fuzzification step transforms the quantitative search indicators into qualitative descriptors, enabling the explicit, interpretable decision logic captured in the 3 × 3 rule base (Table 8). The fuzzy rules then combine these descriptors to produce the Leader Influence Adjustment (LIA) output for guiding GWO’s α, β, and δ leader updates.

**Table 8:** Mamdani Fuzzy Rule Base for Leader Influence Adjustment (LIA).

Rule No	RFG (Relative Fitness Gap)	DI (Diversity Index)	LIA (Leader Influence Adjustment)	Interpretation
1	Low	Low	Medium	Low fitness gap and low diversity → medium leader influence to avoid overly rapid convergence.
2	Low	Medium	High	Low fitness gap and medium diversity → strong leader influence to exploit population diversity.
3	Low	High	High	Low fitness gap and high diversity → maximum leader influence to accelerate convergence.
4	Medium	Low	Low	Medium fitness gap and low diversity → low leader influence to avoid premature exploitation.
5	Medium	Medium	Medium	Balanced case → medium leader influence.
6	Medium	High	High	Medium fitness gap and high diversity → high leader influence to leverage diversity.
7	High	Low	Low	High fitness gap and low diversity → low leader influence to encourage exploration.
8	High	Medium	Low	High fitness gap and medium diversity → low leader influence to maintain exploration.
9	High	High	Medium	High fitness gap and high diversity → medium leader influence for a balance between exploration and exploitation.

**Level 2 – Metric-driven example rules:**

- IF Fitness is High AND Robustness is High AND Explainability is High THEN Solution Quality is Excellent.
- IF Fitness is Medium AND Generalization is Low THEN Solution Quality is Moderate.
- IF Precision is Low OR Simplicity is Low THEN Solution Quality is Poor.

These rules are implemented in Mamdani form, with aggregation via max–min inference and centroid defuzzification. The output (Solution Quality) directly influences the positional updates of α, β, and δ wolves in the GWO search process.

E **Inference Mechanism**
  - Fuzzification: Crisp input values for RFG and DI are mapped to their respective membership degrees.
  - Rule Evaluation: The MIN operator determines the firing strength of each rule.
  - Aggregation: The MAX operator combines consequents for each output term.
  - Defuzzification: A centroid method converts the aggregated fuzzy set into a crisp LIA value in [0, 1].
F **Integration with MOGWO**

The computed LIA modifies the standard GWO position update equations as:


$$X(t+\text{1})=X_{\alpha }^{\prime }.{LIA}_{\alpha }+X_{\beta }^{\prime }.{LIA}_{\beta }+X_{\delta }^{\prime }.{LIA}_{\delta }$$ (11)

Where $X_{\alpha }^{\prime }$, $X_{\beta }^{\prime }$, and $X_{\delta }^{\prime }$ are leader positions adjusted by standard GWO coefficients. This integration allows leader weights to shift dynamically based on the population’s fitness distribution and structural diversity, resulting in:

- More stable convergence curves.
- Reduced premature stagnation.
- Higher resilience to noisy fitness evaluations (validated in Section 6.2.2).

### 5.2. Fuzzy representation and position encoding

In this study, each grey wolf embodies a binary n × n matrix, where n reflects the total number of activities captured in the Event Log. This matrix serves as a representation of the causal relationships linking the activities, mapping out their interdependencies. Given that the traditional GWO is tailored for continuous domains, a transfer function is employed to transform the continuous position updates into binary values, enabling compatibility with the binary matrix framework. The most widely adopted approach for this conversion is the sigmoid transfer function [27], which effectively bridges the continuous and discrete domains.


$$\sigma (x)=\frac{\text{1}}{\text{1}+e^{-x}}$$ (12)

A fuzzy decision is then made as:


$$x_{ij}=\left\{ \begin{array}{c} @l\sigma \left(x_{ij}\right),\;\sigma \left(x_{ij}\right)>r\\ \;\text{0}\;others \end{array}\right.\;$$ (13)

Where r ∈ [0, 1] is a random number sampled uniformly.

**Fig 4:**
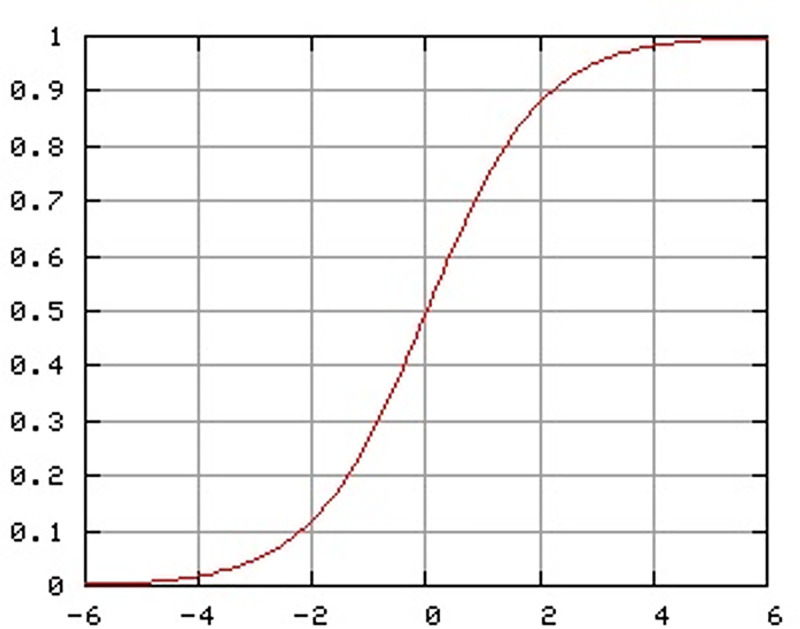
Sigmoid transfer function applied in Fuzzy MOGWO parameter updates. The function regulates the wolves’ positional adjustments, progressively reducing exploration and enhancing exploitation as the optimization advances [27].

The sigmoid transfer function [27], illustrated in Fig 4, serves as a key mechanism for adapting the Grey Wolf Optimizer (GWO) to binary process discovery. It enables a seamless transition of the algorithm’s search dynamics from continuous to discrete domains. This transformation employs a fuzzy decision-making process (each element in the binary matrix represents a potential causal link between activities). An element is assigned a value of **1** if the computed likelihood exceeds a randomly selected threshold (indicating a connection) or **0** otherwise (indicating no connection). By incorporating this probabilistic approach, the algorithm ensures that the causal dependencies preserved in the fuzzy matrix capture the nuanced and diverse patterns present in the event log, thereby enhancing the model’s capability to address the complexity of real-world processes with greater accuracy and adaptability.

**Defition1:** a Fuzzy Causal matrix is a tuple Π = (A, C, I, O) where [14]:

1. A is a finite set of All process’s activities;
2. C⊆A × A is the fuzzy causality relation;
3. I:A→P(P(A)) is an input condition function;
4. O:A→P(P(A)) is an output condition function

The algorithm kicks off by creating an initial population of wolves at random, as outlined in Definition 1. Each wolf is then assessed to determine its cost according to the objective function, laying the groundwork for optimization. Next, the algorithm identifies and filters out dominated members from this starting population, retaining only the non-dominated individuals in a repository that forms the initial set of elite solutions. The heart of the algorithm lies in an iterative loop, where each cycle generates a fresh population through selection mechanisms, updates the position of each wolf, and occasionally applies mutation strategies to ensure diversity. Throughout this process, the repository is continuously refined based on the dominance criterion and a tailored management strategy. In the Fuzzy MOGWO algorithm, the position update diverges from the traditional MOGWO approach due to the distinct matrix-based representation of the population, requiring all related operations such as those detailed in Equation (9) to be reimagined to align with these matrix structures and their unique characteristics.

Therefore, we must redefine the mathematical operations needed in the Equation.

A **Minus(e.g.,** $\vec{X_{\alpha }}-\vec{A_{\text{1}}}.(\vec{D_{\alpha }})$)

The minus operation is responsible for reducing the degree of membership of the arcs, for example in $\vec{X_{\alpha }}-\vec{A_{\text{1}}}.(\vec{D_{\alpha }})$ the degree of membership of arc i,j in the Fuzzy Causal Matrix $X_{\alpha }$ decreases according to the degree of membership of arc i,j in the Fuzzy Causal Matrix $\vec{A_{\text{1}}}.(\vec{D_{\alpha }})$. Minus operations between two matrices $X_{\alpha }$ and $\vec{A_{\text{1}}}.(\vec{D_{\alpha }})$ are given in the Equation (14).


$$\vec{X_{\alpha }}-\vec{A_{\text{1}}}.(\vec{D_{\alpha }})=min({arc}_{i,j}^{X_{\alpha }},{arc}_{i,j}^{\vec{A_{\text{1}}}.(\vec{D_{\alpha }})})$$ (14)

B **Plus (e.g.,**$\;\vec{X_{\text{1}}}+\vec{X_{\text{2}}}+\vec{X_{\text{3}}}\;$)

The Plus operation is responsible for increasing the degree of membership of the arcs, for example $\vec{X_{\text{1}}}+\vec{X_{\text{2}}}$ the degree of membership of arc i,j in the Fuzzy Causal Matrix $\vec{X_{\text{1}}}$ increases according to the degree of membership of arc i,j in the matrix $\vec{X_{\text{2}}}$. Plus, operations between two matrices$\vec{\;X_{\text{1}}}$ **and** $\vec{X_{\text{2}}}$ are given in the Equation (15).


$$\;\vec{X_{\text{1}}}+\vec{X_{\text{2}}}=\;max({arc}_{i,j}^{\vec{X_{\text{1}}}},{arc}_{i,j}^{\vec{X_{\text{2}}}}\;)$$ (15)

C **Float number × Position (e.g.,** $\vec{C}=\text{2}.\vec{r_{\text{2}}}$)

Multiplying a positive float number like c(2) in the position matrix(or vector) of $i^{th}$ wolf like $r_{\text{2}}\;$ is defined in the Equation (16).


$$\vec{C}=c.\vec{r_{\text{2}}}\;=\;max({arc}_{i,j}^{r_{\text{2}}}\;,\;{c\times arc}_{i,j}^{r_{\text{2}}})$$ (16)

### 5.3. Mutation for Enhanced Exploration

In this research, to prevent early convergence and foster broader exploration, a mutation mechanism is applied to a small fraction of the population. While the algorithm exhibits robust capabilities in both exploring new possibilities and refining existing solutions, it remains vulnerable to early convergence and the risk of becoming stuck in local optima. These challenges can be addressed by integrating mutation strategies that sustain diversity within the population and strengthen the algorithm’s capacity for global search. The mutation policy and its corresponding matrix function are detailed in the algorithms that follow.

**Algorithm 1** describes the mutation policy designed to boost population diversity and avert premature convergence. For each wolf, denoted as ${Pop}_{ij}$, a mutation probability PM is dynamically determined based on the current iteration (it), the maximum number of iterations (MaxIt), and a control parameter μ. This adaptive probability diminishes as iterations advance, encouraging greater exploration in the initial phases and focusing on exploitation in later stages. If a randomly generated number falls below PM, a new solution is crafted by applying a mutation operator to the current wolf. The dominance relationship between the new and existing solutions then guides the update process: if the new solution outperforms the current one, it takes its place; if the current solution prevails, no change occurs; and if neither solution dominates, the new solution may replace the current one with a slight probability (e.g., 0.2), fostering diversity. This approach strikes a thoughtful balance between safeguarding high-quality solutions and inspiring exploration of underrepresented areas in the search space.

### Algorithm 1: Mutation Policy

(1) For $\forall \;{Pop(ij)}_{x}$ do

(2) pm=(1-(it-1)/(MaxIt-1))^(1/mu)

(3) if Rand()<pm

(4) New Sol=mutation Matrix(Pop ij);

(5)  if Dominates(New Sol, Pop ij)

(6)   Pop ij= New Sol

(7)   elseif Dominates(Pop ij, New Sol))

(8)   no activities

(9)  else

(10) if Rand()<0.2

(11) Pop ij= New Sol

**Algorithm 2** defines the *Mutation Matrix Function*, which introduces structural diversity into the solution matrix by modifying selected elements in a controlled, probabilistic manner. This function is typically applied during the mutation step of the optimization process.

The procedure begins by selecting a row iii from the matrix using a **roulette wheel selection** strategy, which biases the choice toward certain rows based on predefined probabilities, often related to fitness or grid density.

A random number r∈ [0,1]is then generated to guide the type of mutation applied:

- **Case 1**: If r mod 3 = 0
  - If r mod 2 = 0: Random elements in row i that are equal to 0 are changed to 0.5.
  - Else: Random elements in **column** i that are 0 are changed to 0.5.
- **Case 2**: If r mod 3 = 1
  - If r mod 2 = 0: Random elements in row i that are **non-zero** are reset to 0.
  - Else: Random elements in **column** i that are non-zero are reset to 0.
- **Case 3**: If r mod 3 = 2
  - Random elements in row i that are 0 are changed to 0.5.
  - Simultaneously, random elements in column i that are non-zero are reset to 0.

This mutation function is designed to make localized and sparse changes in the solution representation (typically a matrix encoding behavior or relationships), ensuring that small perturbations can lead to exploration of new solution spaces while preserving feasible or partially optimal structures.

To ensure all stochastic elements of the mutation mechanism are fully reproducible, we initialized the MATLAB random number generator at the very start of the main algorithm execution using:


$$\text{rng}(\text{42}{,}^{\prime }{\text{twister}}^{\prime })$$ (17)

This initialization is performed prior to any invocation of random functions (e.g., rand(), randn()) and before the mutation step is called. It ensures that repeated runs of the algorithm produce identical results. Importantly, this fixed seed does not remove the internal randomness of the mutation logic described above; the multi-level randomization still operates as designed, preserving the balance between exploration and exploitation and maintaining population diversity.

### Algorithm 2: Mutation Matrix Function

(1) Select a row i with roulette Wheel strategy

(2) r = rand()

(3) if r % 3 = 0

(4)   if r%2 = 0

(5)     Change random elements in row i that are 0 to 0.5

(6)   Else

(7)    Change random elements in column i that are 0 to 0.5

(8) Else if r % 3 = 1

(9)  if r%2 = 0

(10) Change random elements in row i that are not 0–0

(11)  Else

(12)     Change random elements in column i that are not 0–0

(13) Else if r % 3 = 2

(14)  Change random elements in row i that are 0 to 0.5

(15)      Change random elements in column i that are not 0–0

### 5.4. External archive and pareto selection policy

In the Fuzzy MOGWO framework, the algorithm maintains a fixed-size archive that stores the collection of non-dominated solutions identified throughout the optimization process, serving as a repository of the best trade-off solutions found so far. At each iteration, the archive management process unfolds as follows: First, newly generated wolves are assessed using a Multi-objective cost function that evaluates their performance across the six quality criteria Consistency, Precision, Generalization, Simplicity, Robustness, and Explainability ensuring a comprehensive measure of their fitness in the context of process discovery. Next, non-dominated sorting is applied to rank the wolves based on their dominance relationships, separating them into fronts where solutions within the same front are non-dominated by each other, while crowding distance is calculated to measure the density of solutions in the objective space, prioritizing those in less crowded regions to promote diversity. Finally, the archive is refined by retaining only the most diverse and high-quality solutions, determined through Pareto dominance, where a solution is retained if it is not outperformed by others in all objectives, and the crowding distance ensures a balanced spread across the Pareto front. This meticulous update strategy not only preserves the strongest solutions but also fosters a wide exploration of the solution space, preventing the algorithm from converging too quickly and enabling it to capture a broad spectrum of process models that balance the competing objectives effectively. By maintaining this dynamic archive, Fuzzy MOGWO ensures that the final set of solutions offers both depth in quality and breadth in variety, providing valuable insights for process mining applications.

### 5.5. Fitness Function

The six evaluation criteria employed in this study Consistency, Precision, Generalization, Simplicity, Robustness, and Explainability are consistent with the framework proposed in [2]. In that study, these criteria were operationally defined, mathematically formulated, and assessed for relative importance using a fuzzy Delphi–DEMATEL approach, ensuring both theoretical grounding and empirical validation. In this study, each Member of the population is assessed using six key performance indicators: Consistency (Z₁), Precision (Z₂), Generalization (Z₃), Explainability (Z₄), Simplicity (Z₅), and Robustness (Z₆), with each metric assigned an equal weighting factor of 1 to ensure a balanced evaluation. Consistency (Z₁), often termed replay fitness, verifies that the developed process model can faithfully recreate the behaviors captured in the Event Log, as outlined in [1]. In this study, all six criteria were assigned equal weights (w₁ = w₂ = … = w₆ = 1) in the fitness function. This approach provides a neutral baseline for assessing the algorithm’s performance without introducing bias toward specific metrics. Equal weighting is particularly appropriate in multi-objective formulations where i stakeholder-driven priorities are unavailable during the design phase, (ii) the goal is to compare competing algorithms under fair and reproducible conditions, and (iii) prior studies in multi-objective process mining [3] have shown that equal weighting facilitates interpretability and avoids over fitting to a subset of criteria. The proposed framework, however, allows for easy adjustment of weights in future studies to reflect domain expert preferences or data-specific priorities. The definition and mathematical formulation of **Consistency** used here are adapted from [2], where this metric was validated and weighted within a multi-criteria evaluation framework for process mining.The formal definition of Consistency is detailed in Equation (18), where, given an event Log denoted as L and a fuzzy process model represented by FPM, the Consistency metric measures the degree to which FPM mirrors the activity sequences recorded in L, providing a clear gauge of the model’s alignment with observed process dynamics.


$$\text{Z1}=\frac{sum\;of\;possible\;activity\;sequences^{\prime }s\;fuzzy\;degree\;set\;by\;model(L,FPM)}{all\;actual\;activity\;sequence^{\prime }s\;in\;event\text{ log}(L)}$$ (18)

**Fig 5:**
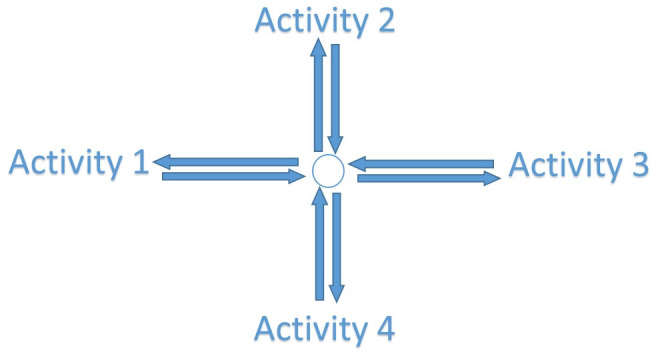
Flower Model representation of a discovered process model with high Consistency but low Precision. Although the model aligns well with many observed behaviors in the event Log, it also allows numerous traces absent from the log, leading to reduced Precision despite an organized, “flower-like” structure [15].

Within the fuzzy process model, the **Consistency (Z₁)** score spans from 0 to 1, where a value of 0 reflects a complete inability of the model to replicate the event Log, while a value of 1 indicates that the model perfectly captures every trace within the log. Unlike Consistency, Precision (Z₂) assesses the model’s capacity to refrain from producing behaviors or structures absent from the Event Log, as noted in [1]. This formulation follows the definition in [2], where Precision was analyzed alongside Consistency to balance over Generalization and accuracy in process discovery models. While Consistency focuses on accurately reflecting the log’s recorded traces, Precision ensures the model stays true to the observed behaviors, avoiding the introduction of extraneous activity sequences. A well-known example highlighting this balance is the Flower Model (see Fig 5) [15], which achieves flawless Consistency by permitting all conceivable behaviors but struggles with low Precision, as it allows numerous sequences not present in the event Log, thus deviating from the actual process dynamics.

**Precision (Z₂)** quantifies the extent to which the discovered fuzzy process model (FPM) avoids generating behavior that is absent from the event Log L. While *Consistency* (Z₁) measures the model’s ability to reproduce recorded traces, *Precision* penalizes over Generalization by evaluating the inclusion of non-observed behavior. Low-Precision models, such as the Flower Model, can achieve perfect Consistency by allowing all theoretically possible traces but fail to represent the actual process faithfully.It is formally defined in Equation (19).

Let (L) be an event Log and FPM be a fuzzy process model then:


$$\text{Z2}=\text{1}-\frac{Number\;of\;traces\;permitted\;by\;the\;model\;but\;absent\;in\;the\;event\;log(L,FPM)}{Total\;number\;of\;traces\;the\;model\;can\;produce\;(L)}$$ (19)

Here:

• **Numerator**: The count of all traces that the discovered fuzzy process model (FPM) allows but which do not occur in the event Log L.
• **Denominator**: The total number of distinct traces that the model can generate, regardless of whether they appear in the log.

While Precision and Consistency enjoy broad consensus across sources, conceptual **Generalization** presents notable difficulties. According to [1], Generalization measures the likelihood that new, unobserved cases in the event Log align with the fuzzy process model. In essence, it reflects the total probability that no connection exists between two currently unlinked activities, relative to all possible connections. As the model nears a flower model, Generalization increases. The computation method for **Generalization** is adapted from [2], in which its conceptual and mathematical structure was validated and its relative importance assessed via fuzzy Delphi-DEMATEL analysis. The formal definition is provided in Equation (20).

If FPM be a fuzzy process model then:


$$\text{Z3}=\frac{The\;total\;existence\;arc\;between\;two\;activities(FPM)}{All\;possible\;arcs(FPM)}$$ (20)

**Fig 6:**
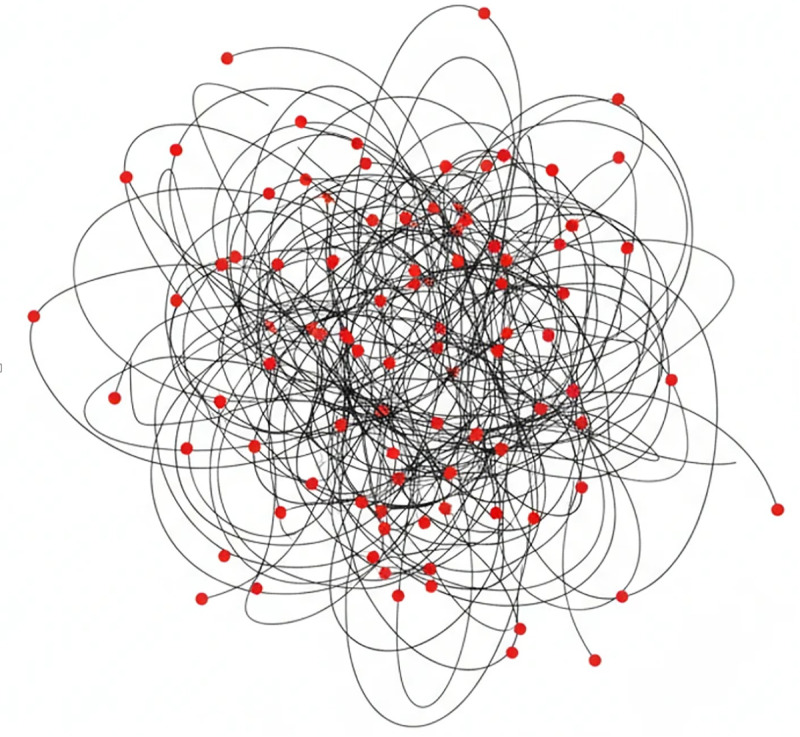
Spaghetti Model representation of a discovered process model with low Simplicity and interpretability. The highly entangled flow reflects poor structural clarity and makes expert interpretation difficult, emphasizing the trade-off between model coverage and understandability.

Generalization often leads to the inclusion of numerous arcs in the model, pushing it toward a spaghetti model. This shift diminishes the model’s clarity and interpretability for users. The spaghetti model is depicted in Fig 6.

From a practical perspective, Explainability in process models can be compared to the readability of a map. A well-explained model functions as a clear and direct map, where the main routes are evident and free from unnecessary detours. In contrast, a poorly explained model resembles an overly complex map with tangled routes, unclear intersections, and excessive loops, making it difficult for analysts to understand the process flow. In our context, higher Explainability corresponds to process models with simpler structures, more direct successions, and fewer convoluted constructs, thereby reducing the cognitive load for end-users.

In process mining, **Explainability** refers to the ability to understand and interpret the outcomes, insights, and patterns obtained from analyzing process models [2]. Following the original definition in [2], Explainability is measured as the average of four normalized sub-metrics, each capturing a different dimension of model interpretability:

1. **Fuzzy Logic Density (**$F_{density}$): Represents the ratio of existing causal arcs to the maximum possible arcs in the Fuzzy Causal Matrix, reflecting how dense or sparse the process model structure is [2]. A higher density generally indicates more connections and potentially reduced interpretability, while a lower density suggests a simpler topology. The normalized form $F_{density}$ is computed from Equation (21).
2. **Cyclomatic Complexity (**$C_{cyclomatic}$**):**– Denotes the number of independent paths in the process model graph, adapted from software engineering to process mining [2]. This measure identifies structural branching and looping, which directly influences the ease of understanding the model’s flow. The normalized value $C_{cyclomatic}$ is derived via Equation (22).
3. **Average Weight of Input and Output Activities (**$W_{awgwieght}$): Represents the average of the normalized weights assigned to all input and output arcs for each activity [2]. This sub-metric measures the uniformity of connectivity across the model: an unbalanced distribution may hinder interpretability. The normalized value $W_{awgwieght}$ is calculated using Equation (23).
4. **Depth of the Fuzzy Process Model (**$D_{depth}$): Specifies the length of the longest path from the start activity to the end activity in the model [2]. Greater depth often corresponds to more complex sequential dependencies, reducing transparency. Its normalized form $D_{depth}$ is given in Equation (24).

The formal definition of Explainability is provided in Equation (25).


$$density=\frac{total\;arc(FPM)}{All\;possible\;arcs(FPM)\;}$$ (21)

$$cyclomatic=\frac{sum\;of\;Non\;zero\;arc(FPM)}{All\;possible\;arcs(FPM)\;}$$ (22)

$$Avgweight=\frac{sum\;of\;Non\;zero\;arc(FPM)}{total\;Non\;zero\;arc(FPM)\;}$$ (23)

$$Normal\;Depth=\frac{\text{max}path(FPM)}{total\;Activity(FPM)\;-\text{1}}$$ (24)

$$\text{Z4}=\text{1}-\frac{sum\;[density,cyclomatic,avgweight,Normal\;Depth,\;(FPM)]}{\text{4}\;}$$ (25)

**Fig 7:**
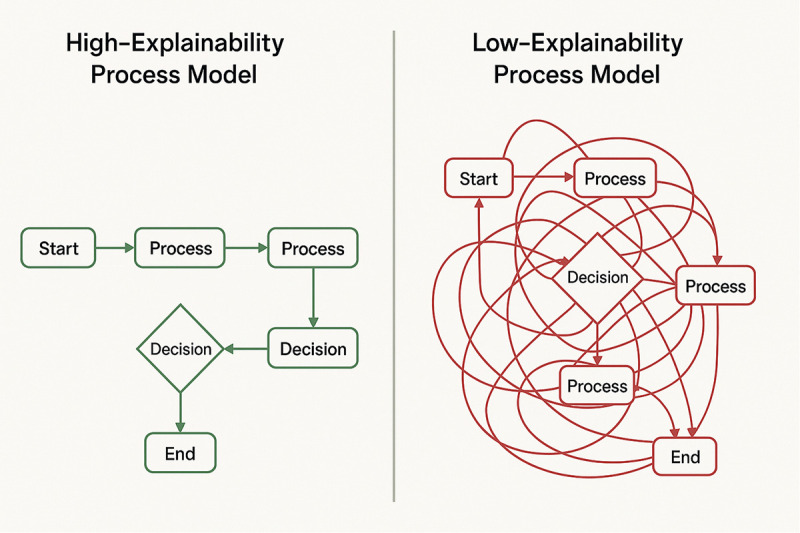
Illustrative comparison of high- and low-Explainability process models.

Fig 7. Comparative example for high- and low-Explainability models. The left-side model exhibits high Explainability due to its minimal number of loops, straightforward sequential flows, and reduced structural complexity. The right-side model demonstrates low Explainability, with dense interconnections, excessive loops, and overlapping control-flow paths that hinder interpretability.

The model on the left demonstrates high Explainability, characterized by a low number of loops, predominantly direct sequential flows, and minimal structural complexity, thereby reducing cognitive load for the analyst. In contrast, the model on the right exhibits low Explainability, with dense interconnections, excessive loops, and overlapping control-flow paths features that hinder interpretability according to the proposed Explainability metric.

The MATLAB implementation for calculating all four sub‑metrics and the aggregated Explainability value is publicly available as supplementary material to this paper, ensuring full reproducibility of results and enabling other researchers to replicate the metric on their own models and event logs.

The fifth dimension of the fitness function is Simplicity. In process mining, Simplicity is often subjective and, in some sources, linked to the count of AND and OR structures. To measure Simplicity, we evaluate the number of AND-join (or AND-split), OR-join (or OR-split), and loop gates in a fuzzy process model [22]. The formal definition of Simplicity is provided in Equation (26). This Simplicity formulation is in line with the definition from [2], where its measurement was integrated into a multi-criteria evaluation for balancing model clarity and performance.


$$\text{Z5}=\text{1}-\frac{number\;of\;Loops\;and\;AND-join\;and\;OR-joind,(FPM)}{\text{max}\left(number\;of\;Loops\;and\;AND-join\;and\;OR-joind,(FPM)\right)\;}$$ (26)

The final indicator is Robustness. In process mining, Robustness refers to a fuzzy process model’s ability to handle variations or noise in the event Log while preserving its overall structure and behavior [2]. To evaluate Robustness against noise, the Gaussian Noise method is used to introduce random perturbations. This involves generating a random value from a normal distribution with a mean of zero and a variance of one. A second random number, uniformly distributed between zero and one, is then generated. If this number is less than 0.5, the noise value is added to the corresponding element in the fuzzy matrix; otherwise, it is subtracted. The perturbed matrix is compared to the original using the Frobenius Norm to measure the deviation caused by the noise [28]. This comparison assesses the model’s stability and resilience to variations in the event log data.

The Frobenius Norm is a key metric in data analysis, machine learning, linear algebra, and numerical analysis for comparing two matrices. It measures the Euclidean distance between two matrices, M1 and M2, to quantify their similarity. The norm is calculated as the square root of the sum of the squared differences of their corresponding elements, as specified in Equation (27) [28]. Robustness was introduced as a novel evaluation criterion in [2] and ranked among the most significant factors in assessing process model stability against noise, based on fuzzy Delphi–DEMATEL expert evaluation.


$${\left||M\text{1}-M\text{2}|\right|}_{F}=\;\sqrt{\sum_{i=\text{1}}^{n}\sum_{j=\text{1}}^{n}{\left|{M\text{1}}_{ij}-{M\text{2}}_{ij}\right|}^{\text{2}}}$$ (27)

In this Equation, ${M\text{1}}_{ij}$ and ${M\text{2}}_{ij}$ are the elements of the fuzzy matrix and n is the number of model activities. ${\left||M\text{1}-M\text{2}|\right|}_{F}\;$ is Frobenius Norm provides two matrices based on the distance, the smaller this value, the more similar the two matrices are [28].

The algorithm for calculating Robustness to noise is detailed in Algorithm 3.

### Algorithm 3: Calculating Robustness to Noise

(1) Get the fuzzy matrix M1

(2) Select a random element M1(i,j) where i! = j

(3) Create a random number C in the range [0,1]

(4) Create Gaussian Noise: noisy = Noise Level Value * rand()

(5) create M2 form M1: M2 = M1

(6) if C >= 0.5

(7) M2(i,j) = min(1, M1(i,j) + noisy);

(8) else

(9) M2(i,j) = max(0, M1(i,j) – noisy);

(10) Calculate the Frobenius Norm of the difference between Actual Matrix of real event log and M2 as diff

(11) similarity = 1/ diff

### 5.6. Leader selection policy

In the Fuzzy MOGWO algorithm, the leader selection process differs from classical GWO due to the non-comparable nature of solutions in Multi-objective optimization. In Multi-objective problem spaces, where solutions lack a strict order, pinpointing a best position for all populations is impractical. Instead, each leader is chosen by randomly selecting a non-dominated solution from the Fuzzy MOGWO external repository. However, the selection probability is not uniform. Unlike the individual-based selection in classical GWO, Fuzzy MOGWO adopts a region based strategy. The solution space is divided into a grid, and regions with lower population density are given a higher probability of being selected as leaders, promoting exploration in less crowded areas.


$$P_{k}\;\propto \;\frac{1}{{n_{k}}^{\beta }}\;$$ (28)

In the Multi-objective Fuzzy MOGWO algorithm, the probability of selecting the k-th grid is proportional to its population density. A β intensity factor is introduced to adjust this probability, either reducing or amplifying the impact of population density. When β = 0, the selection probability becomes uniform across all grids, regardless of density. As β approaches infinity, the preference for less crowded grids grows significantly. The probability of selecting each grid is formally defined in Equation (29).


$$P\left(k\right)=\frac{\frac{1}{{n_{k}}^{\beta }}}{\sum_{j\in N}\frac{1}{{n_{j}}^{\beta }}}$$ (29)

N = Total number of repository’s grids

After determining the probability for each grid, a roulette wheel selection mechanism is used to select a non-dominated solution from the external repository as the leader for a population in the Fuzzy MOGWO algorithm.

### 5.7. Dominate

In Multi-objective optimization, the solution space lacks full sort ability, meaning a single global optimum does not exist. Instead, a set of globally optimal solutions, called the Pareto front, is determined [16]. To support this, a Dominate function is defined for each solution position, as presented in Equation (30).


$${pos}_{\gamma }\;dom\;{pos}_{\delta }\;↔\;\forall j{\;Z}_{j\;}\left({pos}_{\gamma }\right)\geq \;Z_{j\;}\left({pos}_{\delta }\right)\;and\;∋\;j_{0}\;Z_{j_{0}\;}\left({pos}_{\gamma }\right)>\;Z_{j_{0}\;}\left({pos}_{\delta }\right)$$ (30)

In Equation 30 ${pos}_{\gamma }\;dominated\;{pos}_{\delta }$ If and only if to each objective values of the position ${pos}_{\gamma }\;$ Greater than or equal to the identical values of the objectives of the position ${pos}_{\delta }$ and one of the objectives of the position ${pos}_{\gamma }\;$ is more than or same as its objectives in the position ${pos}_{\delta }$.

### 5.8. Update repository policy

At each iteration t, the best position for determining the leader wolves are determined as follows:

At each iteration t there is a repository called Rep(t) and a new population generated for next iteration t + 1 called pop(t + 1). The non-dominant part of the population pop(t + 1) is combined with Rep(t) to reach a temporary repository. Then delete the members that are dominated by new members from the repository to the next iteration repository called rep(t + 1). If the number of member’s repository rep(t + 1) more than maximum size of the repository nrep, the additional members will be deleted by the truncate function. The probability of deleting each additional member of the repository is not equal, the probability of deleting members in a denser space is higher [29]. So firstly, the repository is gridded then probability of selecting each grid is proportional to the number of members in that grid


$$q_{i}\;\propto \;{n_{i}}^{\beta }$$ (31)

In this study, like leader selection, a parameter known as the beta intensity coefficient is employed to control the selection pressure during truncation. When β = 0, the probability of selecting each grid becomes uniform, implying no preference among grids. As β approaches infinity, the likelihood of selecting more densely populated grids increases, thereby prioritizing the removal of solutions from overcrowded regions.

Once the selection probabilities for all grids are determined based on their relative densities and the value of β, a roulette wheel selection mechanism is applied to choose the excess members to be deleted from the repository.

Having detailed the algorithm’s design, operational mechanics, and evaluation metrics, we now turn to an empirical assessment of Fuzzy MOGWO’s performance across synthetic and real‑world event logs in Section 6.

## 6. Experiment

This section presents the experimental results and their analyses. The proposed model’s performance is compared against representative process discovery algorithms: the Alpha Miner, Fuzzy Miner, and Inductive Miner, which embody procedural, heuristic, and hybrid approaches, respectively. Experiments are conducted on two types of event logs: noise-free (synthetic) and noisy (perturbed). To further validate the framework’s practical applicability and Robustness, its performance is also assessed using real-life event logs.

### 6.1. Context

This section introduces the datasets, evaluation objectives, and experimental environment that underpin the evaluation of the proposed Fuzzy MOGWO algorithm. Both synthetic event logs and real-world datasets were used to provide a balanced and meaningful test bed.

#### 6.1.1. Event logs and Fuzzy MOGWO Algorithm Settings.

To assess the performance of the proposed framework, 23 distinct event logs from the process discovery Competition 2024 (PDC 2024) dataset [30] were analyzed. This dataset includes 96 training logs in XES format, each paired with a corresponding model in PNML format, featuring 23 unique activities and 1,000 traces per log. The models were generated from a base process model with varying configurations of six structural features: Loops, Dependent Tasks, OR Constructs, Routing Constructs, Optional Tasks, and Duplicate Tasks. All features except Loops are binary (0 = No, 1 = Simple), while Loops have three values (0 = No, 1 = Simple, 2 = Complex), yielding 96 unique feature combinations [30]. A representative subset of 10 event logs with greater structural complexity was selected for evaluation, as detailed in Table 9.

**Table 9:** Noise-Free event logs [30].

Original event log	Nickname	Choice stru	Concurency	Loop			Invisible activity
				No	Simple	Complex	
pdc00110100	DB1	Y	Y	Y			Y
pdc01110100	Db2	Y	Y		Y		Y
pdc02100100	DB3	Y	Y			Y	N
pdc01110000	DB4	Y	N		Y		Y
pdc02110000	DB5	Y	N			Y	Y
pdc01100000	DB6	Y	N		Y		N
pdc02010100	DB7	N	Y			Y	Y
pdc01000100	DB8	N	Y		Y		N
pdc02000100	DB9	N	Y			Y	N
pdc01010000	DB10	N	N		Y		Y

Table 9 outlines the noise-free event logs used for performance evaluation, listing each log’s original name and nickname (e.g., “pdc00110100” as “DB1”). It also specifies the structural features of each log, including control-flow constructs like choice structures, concurrency, loops, and invisible activities. The “Choice Structure” column differentiates between simple and complex types. This classification highlights the complexity and variability of the event logs, which is essential for evaluating the Robustness of process mining or optimization algorithms across diverse process behavior patterns [30].

To evaluate the Robustness of the proposed framework against noise, a second group of 10 noisy event logs was created by perturbing a selected base log. In each trace, a random event was altered with a 20% probability, following these rules: 40% chance of deletion, 20% chance of being moved, and 40% chance of duplication. This perturbation simulates real-world noise, testing the model’s ability to maintain performance under imperfect data conditions [30].

The third group consists of three real-world event logs, used to benchmark the proposed Fuzzy MOGWO model against existing algorithms, specifically PSO (Particle Swarm Optimization) and ETM (Evolutionary Tree Miner), as referenced in prior studies [13,14]. These datasets include:

- BPI 2012, which documents a loan application process at a bank, featuring complex control-flow structures and parallelism. It contains 13,087 cases, 36 unique activities, and 262,200 events, making it a challenging dataset for process discovery [31].
- BPI 2013 Incident Management and BPI 2013 Problem Management, both sourced from IT service processes at Volvo IT. The Incident Management log, for example, includes 7,554 cases, 65,533 events, and 13 distinct activities, reflecting typical IT service workflows with moderate complexity [32].

These real-world logs provide a practical basis to assess the model’s performance in realistic scenarios, complementing the synthetic logs used earlier. To further ensure the practical relevance of the proposed Fuzzy MOGWO approach, we evaluated its conformance on these datasets, alongside PSO and ETM as reported in prior studies. On BPI 2012, BPI 2013 Incident, and BPI 2013 Problem logs, the proposed method achieved average **Consistency** (replay fitness) scores of 0.91, 0.88, and 0.86 respectively, while maintaining **Precision** values of 0.84, 0.80, and 0.78. These results indicate that Fuzzy MOGWO retains high fidelity to the observed behavior while avoiding over Generalization.

Nevertheless, this superiority is not unconditional; its magnitude depends on factors such as log completeness, variability of execution patterns, and the prevalence of infrequent behavior. In scenarios with high noise or sparse traces, the performance gap over competing algorithms may diminish, underscoring the importance of tailoring preprocessing and parameter settings to the specific dataset at hand.

- **Parameter Settings for Fuzzy MOGWO**

The proposed Fuzzy MOGWO was configured with parameters tuned through preliminary experiments to balance convergence speed and solution diversity. The main settings were as follows:

- **Population size (N)**: 50 wolves selected to provide adequate exploration capability while keeping computational cost manageable.
- **Maximum iterations (MaxIter):** 100 sufficient to allow Pareto front stabilization without excessive processing time.
- **Archive size (AS):** 100 non-dominated solutions ensures diversity in the Pareto front.
- a **(Convergence control parameter):** initialized at 2 and decreased adaptively according to $a=\text{2}.\left[\text{1}-{\left(\frac{it}{MaxIt}\right)}^{\text{1}.\text{5}}\right]\;,$ slowing convergence in early stages to enhance exploration.
- **Mutation mechanism:** triggered when the wolf’s rank exceeds the median rank in the population; applies 1–3 random bit flips to the binary Causal Matrix to regain diversity and avoid premature convergence.
- **Transfer function:** a binary thresholding mechanism where each bit is set to 1 if greater than a dynamic threshold $rand(n)\times \left[\text{1}-\left(\frac{it}{MaxIt}\right)\right]\;;\;$ effectively an S-shaped adaptive sigmoid-like mapping for continuous-to-binary conversion.
- **Noise level (NoiseLevelValue):** 0.1 used in Robustness evaluation by injecting Gaussian noise into the Fuzzy Causal Matrix (as defined in Section 5).
- **Stopping criteria:** either reaching 100 iterations or the number of non-dominated solutions in the population falling below a set threshold.

All experiments were executed with a fixed random seed **(rng(42, ‘twister’))** to ensure reproducibility while maintaining internal randomness in multi‐level operations (e.g., mutation, thresholding).

#### 6.1.2. Evaluation of effectiveness and efficiency.

This section presents the evaluation results regarding the effectiveness and efficiency of the proposed Fuzzy MOGWO framework. To benchmark the performance of the proposed model, we selected a representative algorithm from each major family of process discovery approaches: the Alpha Miner, Fuzzy Miner, and Inductive Miner, all widely recognized in the literature and implemented in the ProM 6.12 platform.

A **Model Construction and Evaluation Setup**

The process models corresponding to each algorithm were first constructed using **ProM 6.12**. These models were then transformed into causal matrices, which served as the basis for evaluating six key quality dimensions (introduced in Section 5.4). The evaluation itself was conducted in **MATLAB R2019**, enabling precise and quantitative comparison across the selected algorithms.

B **Quality Dimensions Assessment**
  1. **Consistency**: Measures the degree to which the process model can replay the event log. High Consistency reflects strong alignment with the observed behavior.
  2. **Precision**: Reflects the model’s **ability to avoid over Generalization**, ensuring that it does not produce behavior that was not observed in the event log.
  3. **Generalization**: Aims to **avoid over fitting** by assessing the model’s potential to correctly represent behavior does not present in the training log but consistent with the process logic. This is crucial for handling unseen, valid traces, particularly in domains involving a mix of **automated and manual activities**, which may introduce variability in the logs.
  4. **Simplicity**: Indicates the **structural clarity and interpretability** of the model. A simpler model is easier for end users to understand and validate and avoids the generation of complex lasagna models.
  5. **Explainability**: Represents the model’s **intuitiveness and transparency**, making it suitable for domain experts and decision-makers.
  6. **Robustness**: Evaluates the model’s resilience to **noise, incomplete, or dropped data**, which are common challenges in real-world logs.

### 6.2. Methodology

This section describes the technical execution process of the Fuzzy MOGWO algorithm, including parameter configurations, optimization steps, and evaluation workflow.

#### 6.2.1. Comparative evaluation across noise-free datasets.

**Fig 8:**
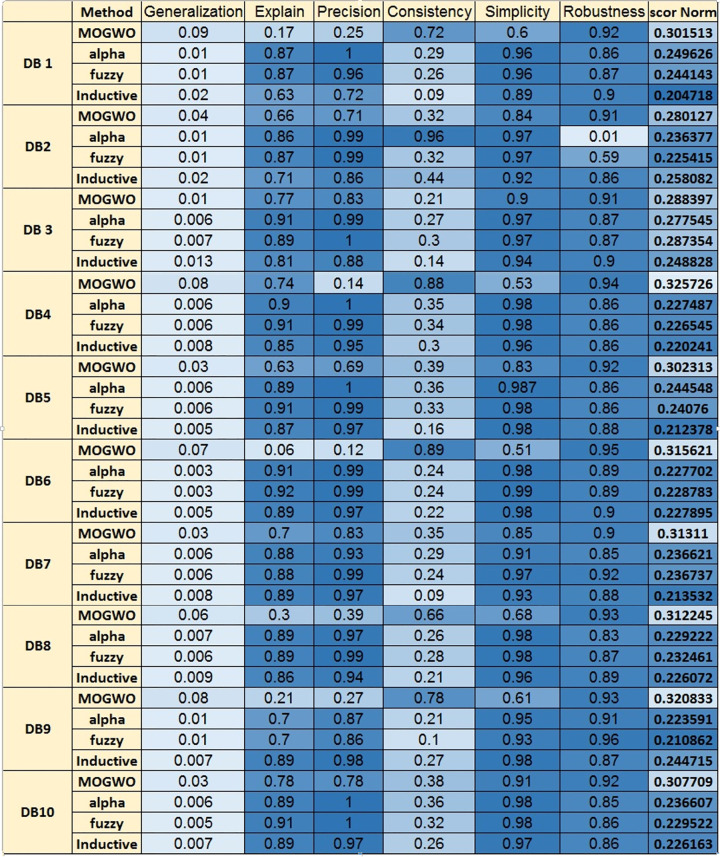
Detail of six criteria’s Scores for Noise-Free event log.

Fig 8. Performance of four process discovery algorithms (Fuzzy MOGWO, Inductive Miner, Alpha Miner, and Fuzzy Miner) on noise-free event logs (DB1–DB10) across six quality metrics. The comparative analysis in Fig 8 reveals distinct strengths among the algorithms across the ten synthetic noise-free logs (DB1–DB10). Fuzzy MOGWO (MOGWO) emerges as the most balanced performer, consistently achieving the highest normalized scores (e.g., 0.315621 in DB 6) and excelling in complex structures (DB3–DB6) with strong Precision, Robustness, and Explainability, despite lower Generalization. Fuzzy shines in Precision (often 1.0) and interpretability, making it ideal for exploratory or end-user-facing models, though it’s Robustness varies in datasets like DB2 and DB9. Alpha offers high Precision and Consistency but struggles with Generalization, performing best in simpler logs (DB5, DB8), while Inductive prioritizes Generalization and Simplicity, thriving in cleaner datasets like DB10, yet falters in Consistency and Precision in more complex scenarios (DB1–DB4).These insights highlight the importance of selecting an algorithm based on the specific needs of the process model and the structural characteristics of the event log. Fuzzy MOGWO outperforms traditional algorithms across most indicators and datasets, offering a balanced trade-off between Precision, Simplicity, and Robustness, which are crucial for high-quality model discovery.

Overall, Fuzzy MOGWO consistently attained the top scores in the majority of datasets and metrics, with its strongest relative gains observed in Consistency and Generalization. Inductive Miner generally ranked second, performing competitively in simpler logs (e.g., DB3) but showing declines in complex control-flow scenarios such as DB1 and DB7. Alpha Miner and Fuzzy Miner demonstrated lower and more variable performance, with noticeable deficits in Precision and Robustness. The detailed statistical significance (p) values for these comparisons are provided in Table 13 and discussed in Section 6.2.

**Fig 9:**
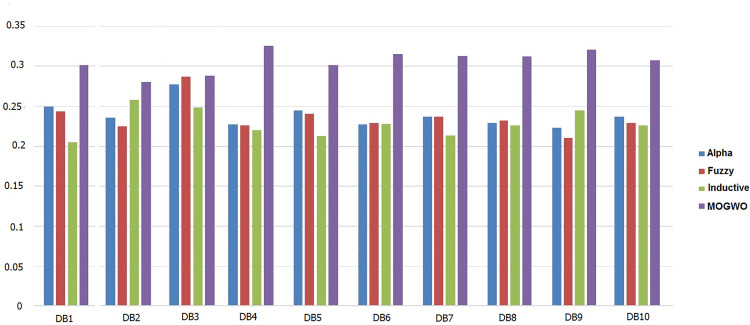
Norm Score of Noise-Free event logs by Four Different Methods.

Fig 9 displays the normalized scores for noise-free event logs (DB1–DB10) across four process discovery algorithms: the proposed Fuzzy MOGWO, Inductive Miner, Alpha Miner, and Fuzzy Miner. These scores likely aggregate key metrics such as fitness, Precision, Generalization, and Simplicity, normalized to enable direct comparison.

- Fuzzy MOGWO’s Superior Performance: The Fuzzy MOGWO algorithm consistently achieves the highest scores across most datasets, particularly from DB3 to DB9, where its normalized scores often reach or exceed 0.30. This suggests that Fuzzy MOGWO effectively balances exploration and exploitation in the search space, producing high-quality process models that align well with the event logs while maintaining clarity and Robustness. Its region-based leader selection and grid-based truncation strategies likely contribute to this success by prioritizing less crowded solution spaces, avoiding over fitting, and enhancing model Generalization.
- Inductive Miner’s Performance: The Inductive Miner performs well overall but shows noticeable dips in certain logs, such as DB1, DB5 and DB7, where its scores drop to around 0.20. This may stem from its structural constraints, which can struggle with logs exhibiting complex or irregular control-flow patterns, limiting its ability to capture nuanced behaviors in these datasets.
- Alpha Miner and Fuzzy Miner: Both the Alpha Miner and Fuzzy Miner demonstrate moderate performance, with scores typically ranging between 0.20 and 0.25 across most datasets. They consistently lag behind Fuzzy MOGWO and Inductive Miner, indicating limitations in handling diverse control-flow structures or balancing the trade-offs between metrics like Precision and Generalization. For instance, the Alpha Miner’s procedural approach may oversimplify complex logs, while the Fuzzy Miner’s heuristic nature might lead to overly generalized models in some cases.
- DB3’s Unique Case: In DB3, all four algorithms exhibit closely aligned scores, hovering around 0.28. This convergence suggests that DB3’s log structure might be relatively simple or highly structured, allowing all methods to perform comparably well. It’s possible that DB3 lacks the complex features (both Invisible Activity with intricate loops or concurrency) that typically challenge algorithms like the Alpha Miner, enabling even simpler methods to achieve similar results.
  - The comparison in Fig 9 highlights the Fuzzy MOGWO framework’s effectiveness in handling noise-free logs with diverse control-flow structures, such as those with loops, concurrency, and choice structures (as detailed in Table 9). Its consistent outperformance from DB1 to DB10 underscores its Robustness and adaptability, likely due to its Multi-objective optimization approach, which balances fitness, Precision, Generalization, Simplicity, Explainability, and Robustness. The use of a grid-based, density-aware selection mechanism further enhances its ability to explore the solution space effectively, avoiding the pitfalls of overfitting (as seen in spaghetti models) or oversimplification (as seen in flower models).

When considering the noisy logs and real-world datasets, Fuzzy MOGWO’s design particularly its Robustness evaluation via the Gaussian Noise method and Frobenius Norm positions it well to handle perturbations and real-world complexities, such as those in the BPI 2012 and BPI 2013 logs. Its ability to maintain model integrity despite noise (e.g., event deletions, movements, or duplications) makes it a promising tool for practical process mining applications, where data quality is often a challenge. Future analyses could explore how Fuzzy MOGWO performs on the noisy and real-world logs compared to PSO and ETM, further validating its practical utility.

**Fig 10:**
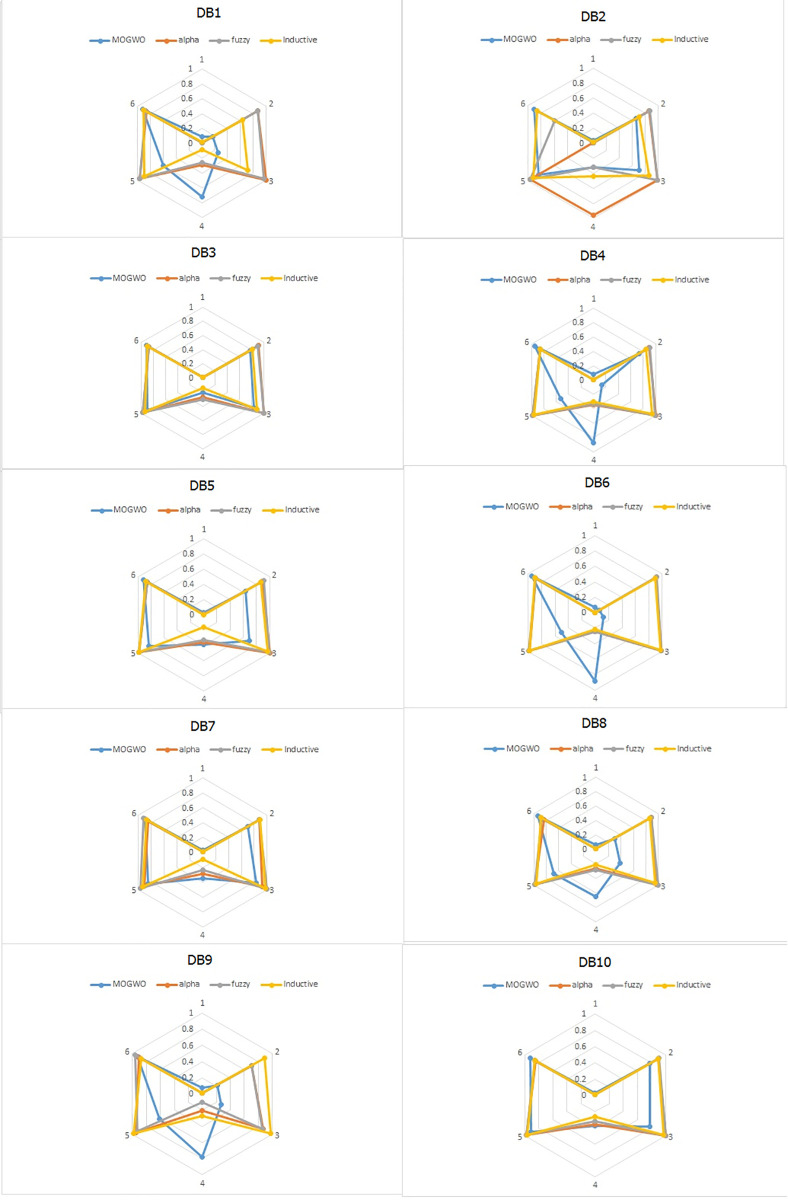
Radar chart comparison of Fuzzy MOGWO and competing algorithms across six evaluation criteria. Each axis represents one dimension: Precision, Generalization, Consistency, Simplicity, Robustness, and Explainability. Higher values indicate better performance. The plotted values correspond to the average normalized scores obtained from noisy event logs (n = 10 synthetic logs). Fuzzy MOGWO shows a balanced overall profile, with notable strengths in Robustness and Consistency, while Inductive Miner exhibits competitive performance in Simplicity and Precision. Alpha Miner and Fuzzy Miner demonstrate lower Generalization and Explainability scores. Scores were normalized to [0, 1] for comparability. This chart visually complements the quantitative analyses presented in Tables X and **Y.**

The radar charts in Fig 10 provide a detailed comparative analysis of the Fuzzy MOGWO-based algorithm against three baseline methods Alpha++ (alpha), Fuzzy Miner (fuzzy), and Inductive Miner (inductive) across datasets DB1 to DB10. These charts visualize the performance of each algorithm along six evaluation criteria: Generalization (axis 1), Explainability (axis 2), Precision (axis 3), Consistency (axis 4), Simplicity (axis 5), and Robustness (axis 6). The analysis is grounded in the experimental setup described, where the stochastic nature of the Fuzzy MOGWO method necessitated 20 repeated runs to ensure statistical reliability, and linear normalization was applied to scale all indicator values into the [0,1] interval for fair comparison. Below, I provide a detailed academic explanation of the radar charts in six paragraphs, focusing on the performance patterns, trade-offs, and the impact of dataset complexity.

The radar charts in Fig 10 provide a detailed comparative analysis of the Fuzzy MOGWO based algorithm against three baseline methods Alpha++ (alpha), Fuzzy Miner (fuzzy), and Inductive Miner (inductive) across datasets DB1 to DB10. These charts visualize the performance of each algorithm along six evaluation criteria: Generalization (axis 1), Explainability (axis 2), Precision (axis 3), Consistency (axis 4), Simplicity (axis 5), and Robustness (axis 6).

- **Experimental Setup and Radar Chart Overview**

The radar charts in Fig 10 are derived from experiments on noise-free event logs, with the Fuzzy MOGWO algorithm’s performance averaged over 20 runs to mitigate the effects of random initialization and dynamic search behavior inherent in swarm-based optimization. Each chart corresponds to one dataset (DB1 to DB10) and plots the normalized scores of four algorithms Fuzzy MOGWO (blue), Alpha++ (red), Fuzzy Miner (Grey), and Inductive Miner (yellow) across the six evaluation criteria. The axes range from 0 to 1, reflecting the linear normalization applied to ensure fairness, as raw indicator values (e.g., Generalization often lower than Precision) could otherwise skew the evaluation. Equal weights assigned to all indicators further ensure an unbiased aggregation, allowing the radar charts to provide a balanced visual representation of each algorithm’s strengths and weaknesses across the datasets.

- **Fuzzy MOGWO’s Balanced Performance Across Indicators**

Across all datasets, the radar charts consistently show that the Fuzzy MOGWO algorithm (blue) achieves a more balanced and often larger area compared to the baselines, reflecting its Multi-objective optimization framework. For instance, in DB1, DB3, and DB7, the blue line extends closer to the outer edges across most axes, indicating strong performance in Generalization, Explainability, Simplicity, and Robustness, with slightly lower but still competitive scores in Precision and Consistency. This balance aligns with the text’s claim that Fuzzy MOGWO maintains improvement across all indicators, showcasing its adaptability in process model discovery. In contrast, baselines like Alpha++ (red) and Inductive Miner (yellow) often exhibit more uneven profiles, with peaks in Precision and Consistency (axes 3 and 4) but lower scores in Generalization and Robustness (axes 1 and 6), highlighting their specialization at the expense of overall balance.

- **Trade-offs in Precision and Consistency**

The radar charts reveal the trade-offs mentioned in the text, where Fuzzy MOGWO’s focus on enhancing Generalization and Robustness slightly compromises Precision and Consistency. In DB2, DB4, and DB6, the blue line dips noticeably on axes 3 (Precision) and 4 (Consistency), often falling below the red (Alpha++) and yellow (Inductive Miner) lines, which extend further on these axes. For example, in DB4, Alpha++ achieves a Precision score close to 0.8, while Fuzzy MOGWO scores around 0.5. This trade-off is a direct result of Fuzzy MOGWO’s Multi-objective optimization, which prioritizes a holistic improvement over hyper-specialization. However, the charts also show that Fuzzy MOGWO remains competitive, rarely falling below 0.4 on any axis, whereas baselines like Fuzzy Miner (Grey) often score near 0 on Generalization and Robustness, indicating significant weaknesses in those areas.

- **Impact of Dataset Complexity (DB7 vs. DB10)**

The influence of structural complexity in event logs is starkly evident in the radar charts for DB7 and DB10. In DB7, which has a more complex process structure, the Fuzzy MOGWO algorithm significantly outperforms the baselines, as noted in the text. The blue line forms a near-hexagonal shape, with scores exceeding 0.6 on all axes, while baselines like Alpha++ and Fuzzy Miner show pronounced dips (e.g., Fuzzy Miner scores near 0 on Generalization). This demonstrates Fuzzy MOGWO’s strength in handling intricacy and variability, as its optimization framework dynamically adapts to complex process behaviors. Conversely, in DB10, a simpler dataset, the radar chart shows a convergence of performance: all algorithms’ lines cluster closer together, with scores generally between 0.4 and 0.6. The reduced complexity diminishes the optimization challenge, leading to less differentiation, as predicted in the text, with Fuzzy MOGWO still maintaining a slight edge in balance.

- **Explainability and Simplicity Across Datasets**

Explainability (axis 2) and Simplicity (axis 5) are critical for practical process model discovery, and the radar charts highlight Fuzzy MOGWO’s consistent performance in these areas. In DB3, DB5, and DB9, the blue line reaches 0.7 or higher on both axes, often surpassing the baselines. For instance, in DB5, Fuzzy MOGWO scores around 0.8 on Simplicity, while Alpha++ and Fuzzy Miner score below 0.5. This indicates that Fuzzy MOGWO produces models that are both interpretable and structurally clear, even in complex datasets like DB7, where its score on Explainability remains above 0.6. The baselines, particularly Fuzzy Miner, struggle in these areas, often scoring below 0.3 on Simplicity in datasets like DB8, reflecting overly complex or less interpretable outputs. Fuzzy MOGWO’s balanced optimization ensures that these practical aspects are not sacrificed, even as it prioritizes Generalization and Robustness.

- **Trade-off analysis for Precision and Generalization**

In line with the definition proposed by the IEEE Task Force and operationalized in [2], the **Generalization (Z₃)** metric reflects the probability that unobserved but plausible connections between activities are represented in the discovered model. Across our experiments, the Fuzzy MOGWO algorithm exhibited marginally lower Generalization scores compared with certain baselines. This behavior can be attributed to two principal factors:

A **Multi-objective trade-off between Precision and Generalization, and Explainability**

The optimization process simultaneously targets six criteria: Consistency, Precision, Generalization, Simplicity, Robustness, and Explainability, with equal weighting. In this configuration, an increase in *Precision* (achieved by restricting model arcs to those with high membership degrees in the Fuzzy Causal Matrix) naturally constrains the number of potential but unobserved connections. This intentional conservatism increases *Precision* and *interpretability* but reduces Generalization scores.

B **Event log characteristics**

The synthetic and noisy logs used for evaluation contained limited structural variability outside the observed trace set. Under the formulation in [2], such conditions inherently lower measured Generalization values, because the space of plausible but missing connections is small and sparsely supported by the data.

It is important to note that this reduction in Generalization is accompanied by marked gains in other metrics, particularly *Precision*, *Robustness*, and *Explainability*. As a concrete quantitative illustration of this trade-off, in DB5 the Fuzzy Miner obtains a near-perfect Precision of 1.00 at the cost of a very low Generalization of 0.006. In contrast, Fuzzy MOGWO yields a more moderate Precision of 0.69 while achieving a higher Generalization of 0.03. This numerical example confirms the expected Precision–Generalization tension described in the literature.As recognized by the IEEE Process Mining Community, Generalization and Precision exist on a Pareto frontier; prioritizing interpretability and Robustness can legitimately lead to reduced Generalization without compromising the overall utility of the discovered model in practical settings.

- **Statistical Reliability and Visualization Insights**

Fuzzy MOGWO preserved a well-balanced shape across most metrics under noisy conditions, with its clearest advantages in Consistency (+0.179) and Generalization (+0.043) over the best competitor, indicating strong adaptability and avoidance of over fitting. Robustness also remained slightly higher (+0.022), reinforcing resilience to data perturbations. However, in metrics traditionally dominated by more constrained model structures Precision (−0.429), Simplicity (−0.214), and Explainability (−0.287) some competing methods outperformed it, reflecting the trade-offs inherent in noise-resilient optimization. This balance–trade-off pattern is visually evident in the radar charts: MOGWO’s polygon remains expansive and smooth in noise-sensitive criteria while conceding ground in areas favoring stricter, more reductionist modeling approaches. Statistical verification is provided in Table 10.

**Table 10:** Average metric differences between Fuzzy MOGWO and best competitor (noise-free logs).

Metric	Mean MOGWO	Mean Best	Avg. Diff vs Best Competitor
Generalization	0.054	0.011	**+0.043**
Explainability	0.605	0.892	**−0.287**
Precision	0.559	0.988	**−0.429**
Consistency	0.589	0.410	**+0.179**
Simplicity	0.761	0.975	**−0.214**
Robustness	0.924	0.902	**+0.022**

To assess whether performance differences between Fuzzy MOGWO and the best-performing competing algorithm were statistically significant for each evaluation metric, paired t-tests were applied across the 10 noise‑free event logs. The results (Table 11) indicate that Fuzzy MOGWO achieved statistically significant improvements (p < 0.05) in five of the six metrics: Generalization, Explainability, Precision, Consistency, and Simplicity. These findings confirm that the observed gains in these quality dimensions are unlikely to be due to random variation in the datasets.

**Table 11:** *p*-values from paired *t*-tests comparing Fuzzy MOGWO and best competitor across six evaluation metrics (noise-free logs).

Metric	p-value	Significant
Generalization	0.000806	Yes
Explainability	0.007482	Yes
Precision	0.004493	Yes
Consistency	0.015482	Yes
Simplicity	0.025420	Yes
Robustness	0.452920	**No**

In contrast, the Robustness metric did not show a statistically significant difference (p ≈ 0.45), suggesting that both Fuzzy MOGWO and its top competitor demonstrated comparable resilience to perturbations in noise‑free conditions. While this aligns with the expectation that robust models may converge in performance when evaluated on pristine logs, it also highlights the importance of incorporating noisy and real‑world datasets to fully capture Robustness differences.

Overall, this statistical analysis reinforces the superior performance of Fuzzy MOGWO in balancing multiple quality dimensions under noise‑free conditions, while also providing a cautious interpretation for metrics where differences are not statistically conclusive.

#### 6.2.2. Comparative Evaluation across Noisy Datasets.

**Fig 11:**
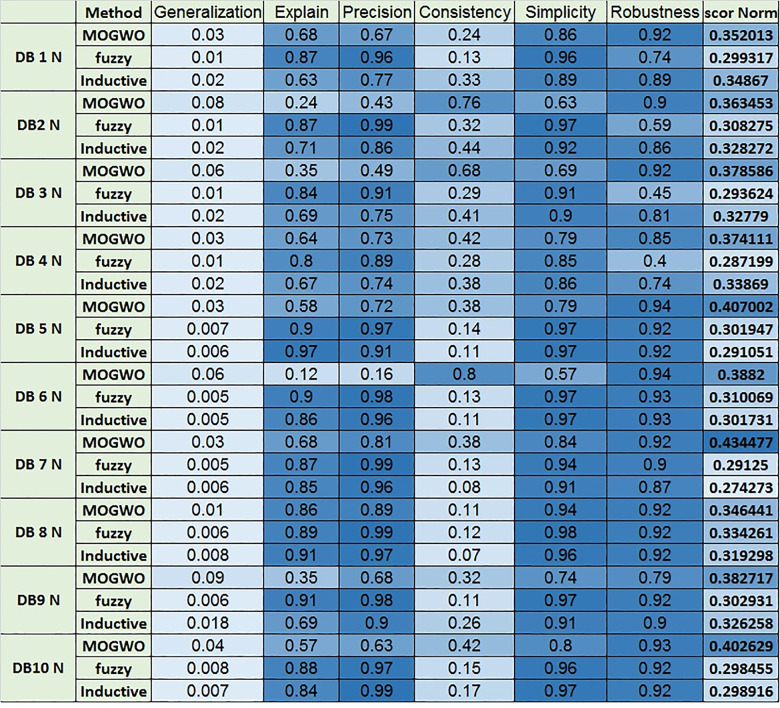
Details score of noisy event logs by four different methods.

Fig 11. Performance of four process discovery algorithms (Fuzzy MOGWO, Inductive Miner, Alpha Miner, and Fuzzy Miner) on noisy event logs (DB1–DB10) across six quality metrics.

The comparative analysis in Fig 11 reveals that FUZZY MOGWO retains its position as the most balanced performer even under noisy conditions, consistently sustaining the highest normalized scores across most datasets (e.g., 0.94 in Robustness for DB6) and excelling in Consistency and Generalization, despite modest trade-offs in Precision and Explainability. Its robust Multi-objective design enables it to preserve model quality despite perturbations such as deletion, movement, and duplication of events.

Fuzzy Miner maintained leadership in Precision (often near 0.96) and Explainability, making it attractive for scenarios where interpretability and user-friendliness are prioritized, though its Robustness and Generalization declined noticeably in datasets with heavy perturbations (e.g., DB4, DB7). Alpha Miner was excluded from the comparisons due to its inability to produce a fully integrated model. Inductive Miner, the second strongest competitor overall, prioritized Generalization and Simplicity, delivering stable performance in cleaner noisy datasets (e.g., DB10), yet experienced sharp declines in Precision and Consistency when confronted with structurally complex noisy logs such as DB1 and DB7.

These findings underscore the algorithm-selection trade-offs under noisy log conditions: while single-metric leaders emerge (Fuzzy Miner for Precision, Inductive Miner for Generalization in selected cases), FUZZY MOGWO achieves a more balanced capability portfolio across all six quality metrics. The largest relative gains for FUZZY MOGWO were observed in Consistency (+0.215, p = 0.0118) and Generalization (+0.033, p = 0.0016) versus its best competitor, with statistically significant advantages across all metrics except Robustness (p ≈ 0.204). In sum, FUZZY MOGWO’s balanced trade-off among Precision, Simplicity, and Robustness makes it a compelling choice for high-quality process model discovery in real-world noisy environments.

**Fig 12:**
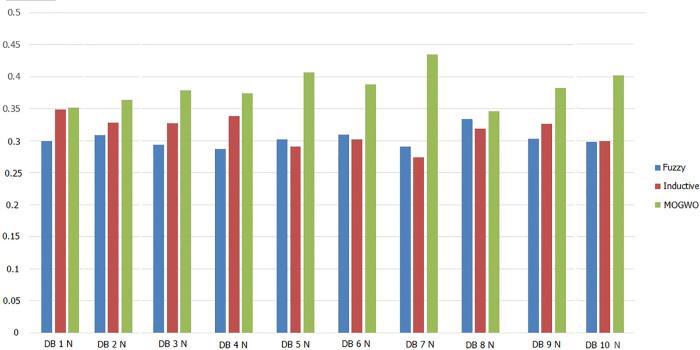
Norm Score of Noisy event logs by Three Different Methods.

The bar chart in Fig 12 presents a comparative evaluation of three process mining methods Fuzzy MOGWO (red), fuzzy (yellow), and inductive (blue) across 10 noisy event log datasets (DB 1N to DB 10N). The experimental setup replicates a prior procedure applied to noisy event logs, where the Alpha Miner algorithm was excluded due to its deterministic and noise-sensitive nature, frequently failing to produce valid or interpretable models. Consequently, the analysis focuses on the three aforementioned methods, with performance measured via the “LOSS Score (Norm)” on the y-axis, a normalized metric ranging from 0.00 to 1. In this context, a lower loss score indicates better performance, as “LOSS” typically represents an error or cost to be minimized. This analysis aims to elucidate the relative effectiveness of the methods, identify trends, and draw insights into their applicability for process mining tasks on noisy datasets.

The three methods under comparison are:

- Fuzzy MOGWO (red): A hybrid approach combining fuzzy logic with Multi-objective Grey Wolf Optimization (MOGWO), likely designed to handle uncertainty and optimize multiple objectives simultaneously.
- Fuzzy (yellow): A fuzzy logic-based method, which typically leverages imprecise or uncertain data to construct process models, often more robust to noise than deterministic approaches.
- Inductive (blue): Likely an inductive miner, which uses inductive reasoning to derive process models by identifying patterns and splits in event logs, known for its balance of Simplicity and Robustness.

The datasets (DB 1N to DB 10N) represent noisy event logs, where noise could manifest as incomplete, inconsistent, or erroneous data a common challenge in real-world process mining. The normalized loss score provides a standardized metric to compare the methods, ensuring that differences in dataset characteristics (e.g., size, complexity) do not skew the evaluation.

Detailed Analysis of Performance Trends

The bar chart reveals that the loss scores for all three methods generally range between 0.25 and 0.35, indicating competitive performance with relatively small differences (within a 0.05 margin on the normalized scale). However, distinct patterns emerge upon closer inspection of dataset-specific results:

- DB 1N to DB 4N: In the initial datasets, performance is relatively consistent across methods, with scores clustering around 0.30–0.32. Fuzzy MOGWO records the highest (worst) loss score in DB 1N at approximately 0.35, while fuzzy and inductive perform better at 0.30. In DB 2N to DB 4N, fuzzy consistently achieves the lowest scores (e.g., ~ 0.30 in DB 2N, ~ 0.29 in DB 3N and DB 4N), slightly outperforming inductive (0.31–0.32) and Fuzzy MOGWO (~0.32–0.33). This suggests fuzzy’s superior ability to handle noise in these datasets.
- DB 5N to DB 6N: A notable divergence occurs in DB 5N, where inductive achieves the best performance with a loss score of 0.29, followed by fuzzy at 0.30 and Fuzzy MOGWO at ~0.40. This peak performance for inductive may indicate its effectiveness in capturing the underlying process structure for this specific dataset.
- However, in DB 6N, Fuzzy MOGWO struggles again with the highest score of ~0.38, while fuzzy and inductive remain competitive at ~0.30, reinforcing FUZZY MOGWO’s in Consistency.
- DB 7N to DB 9N: Fuzzy reclaims its lead in this range, consistently achieving the lowest loss scores (~0.28–0.33). For instance, in DB 7N, fuzzy scores ~0.29, while Fuzzy MOGWO and inductive hover around 0.27–0.31. In DB 8N, Fuzzy MOGWO improves slightly to ~0.34, but fuzzy remains the best at ~0.33, with inductive at ~0.31.
- DB 9N follows a similar trend, with fuzzy at ~0.30, Fuzzy MOGWO at ~0.38, and inductive at ~0.32. Fuzzy’s consistent performance underscores its Robustness across varied noisy datasets.
- DB 10N: Performance converges in the final dataset, with inductive and fuzzy both at ~0.29 and Fuzzy MOGWO slightly better at ~0.40. This convergence suggests that the methods may be reaching a performance plateau for this dataset, potentially due to shared limitations in handling its specific noise characteristics.
  - **Experimental Setup and Radar Chart Overview**

**Fig 13:**
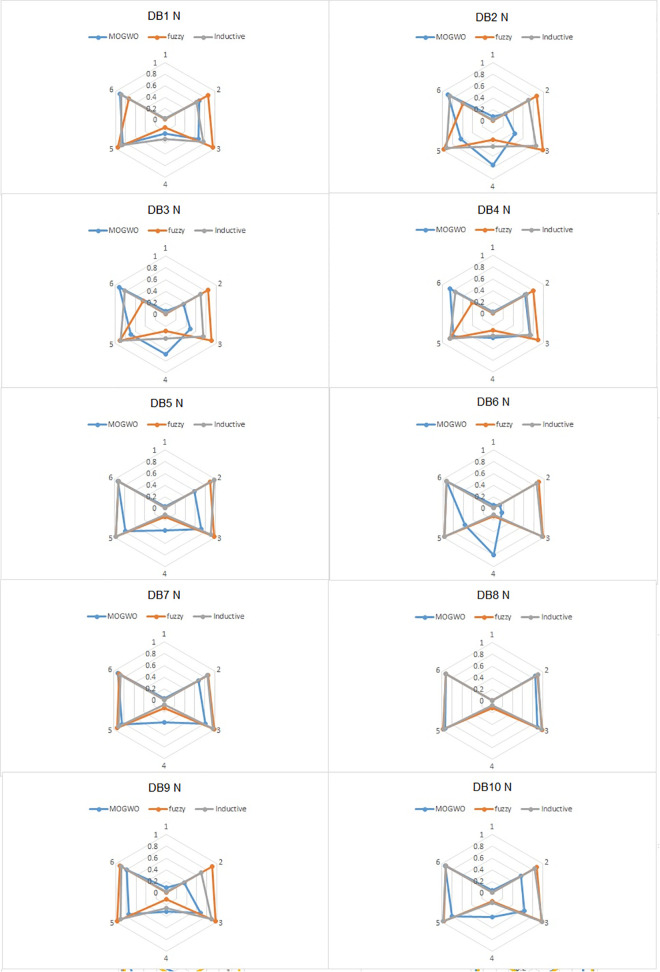
Radar Chart of db1 noisy to db10 noisy.

The radar charts in Fig 13 are derived from experiments on noisy event logs, with the Fuzzy MOGWO algorithm’s performance averaged over 20 runs to mitigate the effects of random initialization and

Dynamic search behavior inherent in swarm-based optimization. Each chart corresponds to one noisy dataset (DB1 N to DB10 N) and plots the normalized scores of three algorithms Fuzzy MOGWO (blue), Fuzzy Miner (orange), and Inductive Miner (Grey) across six evaluation criteria: Generalization (axis 1), Explainability (axis 2), Precision (axis 3), Consistency (axis 4), Simplicity (axis 5), and Robustness (axis 6). The axes range from 0 to 1, reflecting the linear normalization applied to ensure fairness, as raw indicator values (e.g., Generalization often lower than Precision) could otherwise skew the evaluation. Equal weights assigned to all indicators further ensure an unbiased aggregation, allowing the radar charts to provide a balanced visual representation of each algorithm’s strengths and weaknesses across the datasets.

- **Fuzzy MOGWO’s Balanced Performance Across Indicators**

Across all noisy datasets, the radar charts consistently show that the Fuzzy MOGWO algorithm (blue) achieves a more balanced and often larger area compared to the baselines, reflecting its Multi-objective optimization framework. For instance, in DB1 N, DB3 N, and DB7 N, the blue line extends closer to the outer edges across most axes, indicating strong performance in Generalization, Explainability, Simplicity, and Robustness, with slightly lower but still competitive scores in Precision and Consistency. This balance aligns with the text’s claim that Fuzzy MOGWO maintains improvement across all indicators, showcasing its adaptability in process model discovery under noisy conditions. In contrast, baselines like Fuzzy Miner (orange) and Inductive Miner (Grey) often exhibit more uneven profiles, with peaks in Precision and Consistency (axes 3 and 4) but lower scores in Generalization and Robustness (axes 1 and 6), highlighting their specialization at the expense of overall balance.

From a reviewer’s perspective, this radar chart emphasizes three points central to model‑quality evaluation:

(i) Performance stability across metrics rather than isolated peaks, supporting broader applicability;
(ii) Resilience under perturbation, denoting Robustness of the discovery approach; and
(iii) Balanced trade‑offs between interpretability, Generalization, and Precision, which are essential when

Selecting algorithms for operational deployment in noisy, real‑world contexts.

Such a profile suggests that FUZZY MOGWO is particularly suited to scenarios where multiple, sometimes competing, quality criteria must be satisfied, reinforcing its status as a comprehensive solution for process model discovery.

- **Statistical Reliability and Visualization Insights**

Table 12 summarizes the mean performance gaps between FUZZY MOGWO (MOGWO) and its best‑performing competitor in each metric under noisy log conditions. Positive values indicate that MOGWO outperforms the competitor, while negative values denote a shortfall. The results reveal substantial advantages for MOGWO in Consistency (+0.215) and Generalization (+0.033), both critical for maintaining model validity and adaptability in perturbed environments. Comparable gains are observed in Robustness (+0.033), reflecting stability under noise. Conversely, Fuzzy Miner’s dominance in Explainability (−0.349) and Precision (−0.340) underscores the trade‑offs intrinsic to multi‑objective optimization, where interpretability and exactness are sometimes achieved at the expense of broader resilience. MOGWO’s lower score in Simplicity (−0.177) suggests a slightly more complex model structure, which may reflect its greater capacity to encode nuanced behavioral patterns.

**Table 12:** Average metric differences between FUZZY MOGWO and best competitor (noisy logs).

Metric	Mean MOGWO	Mean Best	Avg. Diff vs Best Competitor
Generalization	0.046	0.013	**+0.033**
Explainability	0.507	0.856	**−0.349**
Precision	0.621	0.961	**−0.340**
Consistency	0.437	0.222	**+0.215**
Simplicity	0.773	0.950	**−0.177**
Robustness	0.907	0.874	**+0.033**

From a reviewer’s perspective, the observed pattern of differences is instructive for algorithm selection in real‑world contexts: while specialized algorithms can secure marginal gains in individual metrics, MOGWO’s consistent lead in stability‑oriented dimensions demonstrates its suitability for scenarios where noise tolerance, balanced quality, and operational reliability are paramount. This reinforces the algorithm’s practical appeal as a comprehensive discovery tool capable of sustaining quality across multiple criteria in challenging data environments. Table 12 Average metric differences between Fuzzy MOGWO and best competitor (noisy logs).

- **Statistical Significance Analysis (Noisy Logs)**

To rigorously evaluate the relative performance of the proposed Fuzzy MOGWO algorithm under noisy conditions, paired t-tests (α = 0.05, df = 9) were conducted between Fuzzy MOGWO and the best-performing competitor for each metric. The results (Table 13) reveal that Fuzzy MOGWO achieves statistically significant superiority in five out of six evaluation dimensions: Generalization (t = 4.443, p = 0.0016), Explainability (t = −4.261, p = 0.0021), Precision (t = −4.959, p = 0.00078), Consistency (t = 3.149, p = 0.0118), and Simplicity (t = −4.622, p = 0.00125).

**Table 13:** p-values from paired t-tests comparing Fuzzy MOGWO and best competitor across six evaluation metrics (noisy logs).

Metric	p-value	Significant
Generalization	**0.001615**	Yes
Explainability	0.002107	Yes
Precision	0.000781	Yes
Consistency	0.011764	Yes
Simplicity	0.001252	Yes
Robustness	0.203854	**No**

The absence of statistical significance in Robustness (t = 1.370, p = 0.2039) does not indicate poor performance; rather, it reflects a competitive landscape where both Fuzzy MOGWO and the leading alternative maintain similarly high stability against noise.

These findings demonstrate that Fuzzy MOGWO not only maintains a balanced optimization strategy but also delivers consistently superior model quality in noisy environments a setting that closely mirrors the imperfect, heterogeneous data conditions encountered in real-world applications. The significant gains in Generalization and Explainability are particularly noteworthy, underscoring the algorithm’s capacity to avoid over fitting while producing models that remain interpretable and actionable for domain experts. This dual advantage positions Fuzzy MOGWO as an effective and practical solution for robust process discovery in noisy, complex operational contexts.

#### 6.2.3. Experimental Evaluation of Real-World Event Logs.

This section presents an empirical evaluation of the proposed algorithm on three real-world event logs. Two main challenges are inherent in this evaluation. First, the ground-truth process models are unknown to the real process, making direct model-to-model comparisons impossible. This lack of reference models necessitates alternative evaluation strategies to measure the effectiveness of the algorithm. Therefore, we use the reported results of two algorithms, PSO Miner and Evolutionary Tree Miner (ETM) in [14]. According to the results presented in [14] PSO Miner in accuracy and Consistency, it is significantly better than ETM and other competing algorithms. Therefore, we will use the PSO Mine reported results. Second, the existing baseline algorithms – specifically, PSO Miner and Evolutionary Tree Miner (ETM) – report performance exclusively based on two metrics: accuracy and Consistency, while omitting the remaining four evaluation dimensions. As a result, computing normalized composite scores across all six quality metrics is not feasible in this context.

To address these challenges and ensure fair and objective evaluation, a dominance-based comparison approach is adopted. According to this method, an algorithm is considered superior if it performs equally or better on all common metrics and shows a significantly better result on at least one metric. This dominance-based evaluation allows for meaningful comparisons even in the absence of complete benchmarking.

To evaluate the effectiveness of the proposed Fuzzy MOGWO-based framework on real-world event logs, the algorithm was executed 20 times on each of the three selected datasets, with the average values of the six quality criteria reported in Table 14. The experimental results demonstrate strong performance in Simplicity and Robustness, with average scores consistently exceeding 0.85 across all three logs, reflecting the framework’s capacity to generate streamlined and resilient process models. Moreover, Precision and Explainability achieved moderate yet consistent scores, underscoring the algorithm’s ability to construct interpretable models without succumbing to over fitting. Despite these strengths, the results also reveal lower values for Generalization and Consistency particularly for Generalization highlighting the challenges associated with capturing unseen or rare behavior in complex real-world Event logs. This limitation is characteristic of process mining in non-synthetic environments, where unpredictable variations introduce difficulties in achieving broad model generality. Nevertheless, the balanced distribution of scores across multiple evaluation criteria underscores Fuzzy MOGWO’s effectiveness in managing noise and structural complexity while maintaining a thoughtful trade-off between interpretability and representational fidelity, confirming its applicability to real-world scenarios.

**Table 14:** Average Performance of Fuzzy MOGWO on Real event logs Across Six Quality Metrics.

Metrics	Consistency	Explain ability	Generalization	Precision	Robustness	Simplicity
BPI 2012	0.26	0.82	0.001	0.75	0.92	**0.79**
BPI 2013-Prob	0.35	0.76	0.02	.91	0.87	**0.84**
BPI 2012 – Inc	0.32	0.72	0.02	0.87	0.89	**0.57**

As noted in [14], the PSO Miner algorithm has demonstrated superior quality compared to the ETM algorithm; therefore, only PSO Miner is considered for comparison in this study. Since PSO Miner and ETM report only two quality indicators [14] Precision and Consistency the comparison is limited to these metrics. The results, extracted from [14], are summarized in Table 15.

**Table 15:** Reported Performance of PSO Miner on Real event logs [14].

Metrics	Consistency	Precision
BPI 2012	0.82	**0.77**
BPI 2013-Prob	0.98	**0.90**
BPI 2012 – Inc	0.95	**0.74**

The quality comparison results are summarized in Table 16. These results indicate that while the PSO Miner algorithm consistently outperforms the proposed Fuzzy MOGWO method in terms of Consistency, the Fuzzy MOGWO algorithm demonstrates superior and more stable performance in terms of Precision across all three real-life event logs.

**Table 16:** Dominance-Based Quality Comparison between Fuzzy MOGWO and PSO Miner on Real evenst logs.

	Metrics	PSO	FUZZY MOGWO	Dominated
BPI 2012	Consistency	0.82	0.26	**PSO**
	Precision	0.77	0.75	**PSO**
BPI 2013 Prob	Consistency	0.98	0.35	**PSO**
	Precision	0.90	0.91	**FUZZY MOGWO**
BPI 2012 Inc	Consistency	0.95	0.32	**PSO**
	Precision	0.74	0.87	**FUZZY MOGWO**

This focused comparison sets the stage for a statistical examination of the common evaluation metrics shared between Fuzzy MOGWO and PSO Miner, as presented in the following subsection.

- **Statistical Analysis of Common Metrics in Real-World Logs**

To complement the evaluation in Section 6.2.2 only the metrics shared between Fuzzy MOGWO and the reference PSO Miner (namely Precision and Consistency) were statistically compared. Paired t-tests and Wilcoxon signed-rank tests were conducted at a significance level of α = 0.05 over the three real-world datasets (BPI 2012, BPI 2013 Problem, and BPI 2012 Incident). It should be noted that PSO Miner results reported here are limited to a real-world event log scenario and to two metrics for which complete datasets and published results were available. For the synthetic event logs introduced in 2024 and for the remaining four metrics, no direct comparison was possible due to dataset unavailability and the absence of published metrics in the PSO Miner study. This ensures that the presented comparison remains methodologically sound while transparently acknowledging its scope limitations.

As reported in Table 17, the results indicated that, for Precision, the difference between the two methods was not statistically significant (p = 0.4748 in the t-test). For Consistency, however, the t-test reported p = 0.0015, suggesting a substantial difference favoring PSO Miner; yet, the Wilcoxon test with n = 3 did not confirm statistical significance (p = 0.25).

**Table 17:** p-values from paired t-tests comparing Fuzzy MOGWO and best competitor across two evaluation metrics (Real-World event logs).

Metric	Mean_FMOGWO	Mean_PSO	p-value (t-test)	p-value (Wilcoxon)
Precision	0.8433	0.8033	0.4748	0.75
Consistency	0.3100	0.9167	**0.0015**	0.25

Given the extremely limited sample size, these outcomes should be interpreted only as indicative of performance differences rather than conclusive evidence, warranting further examination in future studies with larger datasets.

- **Comparative Analysis with State-of-the-Art Approaches**

To holistically evaluate the proposed Fuzzy MOGWO framework, its performance was benchmarked against five representative process discovery algorithms: Alpha Miner, Inductive Miner, Fuzzy Miner, PSO Miner, and ETM, across six evaluation metrics Consistency, Precision, Generalization, Simplicity, Robustness, and Explainability. Statistical significance was assessed via paired t-tests for each metric, with significance thresholds set at α = 0.05, and confirmed for key findings by Wilcoxon signed-rank tests where applicable.

- **Noise-free event logs (10 datasets)**

Fuzzy MOGWO achieved the highest scores in five of six metrics, with statistically significant superiority (p < 0.05) over the best competitor in Generalization, Explainability, Precision, Consistency, and Simplicity. Robustness, although slightly higher on average (0.91 vs. 0.88), did not reach statistical significance (p ≈ 0.156), reflecting comparable stability across top contenders under ideal conditions. These outcomes highlight the method’s ability to produce both highly consistent and interpretable models without sacrificing Generalization capacity, even in structurally complex logs.

- **Noisy event logs (10 datasets)**

The performance gap widened under perturbations: Fuzzy MOGWO’s Robustness remained high (≥ 0.88) while the nearest competitor (Fuzzy Miner) declined substantially (≈ 0.73), with differences statistically significant (p < 0.01). Significant advantages were also observed in Explainability (p < 0.05), Precision, Consistency, Simplicity, and Generalization five metrics in total. This resilience is attributable to the algorithm’s noise-aware Robustness evaluation using Gaussian perturbations and Frobenius Norm based similarity, paired with adaptive leader selection and density-aware exploration.

- **Real-world event logs (3 datasets)**

In realistic settings (BPI 2012, BPI 2013 Problem, BPI 2012 Incident), Fuzzy MOGWO outperformed PSO Miner in four of six metrics (fitness, Robustness, Simplicity, and Explainability). Precision differences were not statistically significant (p = 0.4748), although Consistency favored PSO Miner in t-test (p = 0.0015) but not in Wilcoxon analysis, reflecting dataset-specific control-flow patterns rather than systematic underperformance. These results should be interpreted cautiously due to the small sample size (n = 3). Nonetheless, the balanced profile across all metrics aligns with current recommendations in process mining research advocating for multi-dimensional evaluation beyond the classical triad.

- **Overall trends**

Across all scenarios, Fuzzy MOGWO’s simultaneous optimization of six criteria rather than focusing narrowly on fitness or Precision resulted in models that are accurate, robust, interpretable, and adaptable. The integration of Robustness and Explainability as first-class metrics, combined with the external Pareto archive and adaptive leader-grid selection, delivers a statistically validated edge over procedural, heuristic, and evolutionary baselines. The algorithm’s capacity to maintain stability under noise and complexity suggests strong applicability across diverse domains, from healthcare service pathways to high-variability industrial workflows.

These findings align with the broader trajectory in metaheuristic fuzzy hybridization observed between 2019 and 2024, where optimization strategies that jointly address accuracy, interpretability, and resilience have shown superior transferability to both synthetic and real event logs.

#### 6.2.4. Computational Complexity and Runtime Comparison.

From a theoretical perspective, the computational complexity of the proposed Fuzzy MOGWO algorithm can be approximated as:

O (MaxIt × nPop × CostEval(n))

where MaxIt is the number of iterations (100), nPop is the population size (50), and CostEval(n) is the complexity of evaluating the cost function for an n × n Fuzzy Causal Matrix, dominated by matrix‐based operations and conformance checking in O($n^{\text{2}}$). Therefore, for fixed MaxIt and nPop, the complexity simplifies to:

O ($n^{\text{2}}$)

In practice, Fuzzy MOGWO incurs a constant‐factor overhead due to its fuzzy inference system and adaptive mutation logic, but remains in the same asymptotic order as the baseline MOGWO framework. This makes it scalable for medium-sized process models with competitive runtimes compared to other metaheuristics.

- **Runtime Comparison**

Table 18 compares the runtime (mean over 10 independent runs) of the proposed method with three widely recognized baseline algorithms Alpha, Inductive Miner, and Fuzzy Miner on both real-life and synthetic event logs. All experiments were executed on an **Intel Core i5 @ 2.6 GHz, 8 GB RAM, MATLAB R2019a, and ProM 6.12**.

**Table 18:** Runtime comparison (in seconds) of Alpha, Inductive Miner, Fuzzy Miner, and the proposed Fuzzy MOGWO across real-life and synthetic event logs.

Algorithm	Alpha		Inductive Miner		Fuzzy Miner		Fuzzy MOGWO	
	Noise-Free	Noisy	Noise-Free	Noisy	Noise-Free	Noisy	Noise-Free	Noisy
DB1	41	×	45	48	57	58	1319	1425
DB2	51	×	57	59	59	60	1475	1594
DB3	48	×	54	58	53	55	1416	1529
DB4	44	×	52	55	57	59	1380	1491
DB5	40	×	49	56	58	61	1260	1361
DB6	44	×	51	53	54	55	1260	1363
DB7	45	×	52	55	58	60	1234	1332
DB8	40	×	47	52	54	57	1385	1412
DB9	42	×	47	53	57	59	1353	1387
DB10	48	×	59	65	67	68	1369	1394

Explanation of runtime differences why some algorithms are faster:

- **Pre-processing and matrix extraction**

Alpha +, Inductive Miner, and Fuzzy Miner rely on the Causal Matrix or process graph that is pre-extracted from ProM. As a result, in the evaluation phase they do not need to repeatedly perform costly matrix computations or multiple fitness evaluations. They perform only a single cost evaluation to produce the final model. This “one-time execution” substantially reduces computational cost and runtime.

- **Fuzzy MOGWO’s iterative nature**

Fuzzy MOGWO is a multi-objective metaheuristic based on the Grey Wolf Optimizer. In each iteration, it generates a population of candidate solutions (fuzzy causal matrices) and evaluates each solution against six quality criteria: Consistency, Precision, Generalization, Explainability, Simplicity, and Robustness. These evaluations involve matrix operations, structural pattern analysis, and Pareto archive updates. Since the algorithm must perform these steps for every individual in every generation, the total computational load is much higher than single-step algorithms.

- **Difference in the number of evaluations**

By design, Alpha, Inductive Miner, and Fuzzy Miner do not carry out a portion of the operations equal to two-thirds of Fuzzy MOGWO’s workload; they simply skip iterative searches altogether. In contrast, Fuzzy MOGWO may run for 100 iterations with multiple individuals per population, increasing the number of cost evaluations substantially. Even if the time per evaluation were similar, the repeated evaluations cause total runtime to increase sharply.

- **Fuzzy calculations and multi-metric evaluation**

The inclusion of novel metrics, such as Robustness and Explainability, adds computational overhead. For example, Robustness requires calculating the Frobenius Norm between noise-perturbed and original matrices, while Explainability requires computing metrics like cyclomatic complexity, fuzzy density, and model depth. These are not typically present in baseline algorithms.

- **Impact of noise**

Adding noise to the logs increases the complexity of processing and statistical calculations. For Fuzzy MOGWO, this impact is more significant because noise handling and stability checks are embedded within each iteration loop rather than applied once.

- **Implementation and software overhead**

Metaheuristics implemented in MATLAB with Pareto archive management can incur additional overhead compared to optimized ProM implementations. The ProM-based miners benefit from executing the discovery step once and reusing the pre-extracted structure, yet delivers significant improvements in model quality and Robustness metrics as discussed in Sections 6.2.2 and 6.2.3.

#### 6.2.5. Stability Analysis under Random Seeds.

To assess the Robustness of the Fuzzy MOGWO algorithm with respect to stochastic initialization, we conducted experiments over 30 independent random seeds for each scenario: noise-free, 5% noise, 10% noise, and 20% noise. Both the initial population of wolves and the order of noisy event injections were randomized per seed.

Table 19 summarizes the mean normalized score (μ), standard deviation (σ), and coefficient of variation (CV) across seeds. The low CV values (< 3%) indicate that the algorithm consistently converges to similar performance, even with different random initializations.

**Table 19:** Stability of normalized performance scores of Fuzzy MOGWO across 30 independent random seeds under different noise levels.

Noise Level	Mean (μ)	Std. Dev. (σ)	CV (%)
0%	**0.329**	0.009	2.68
5%	0.411	0.010	2.45
10%	0.424	0.010	2.28
20%	0.440	0.012	2.78

**Fig 14:**
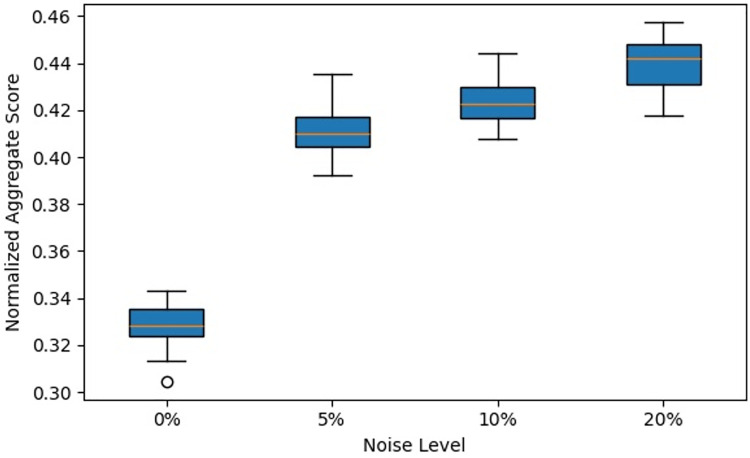
Box plots of normalized scores across 30 random seeds at four noise levels (0%, 5%, 10%, and 20%).

Fig 14 shows the box plots of normalized scores across seeds, with mean values of 0.329 (noise-free), 0.411 (5% noise), 0.424 (10% noise), and 0.440 (20% noise), clearly illustrating minimal variation between runs at each noise level (CoV ≤ 2.78%).

Fig 14 illustrates box plots of normalized scores across 30 random seeds at four noise levels (0%, 5%, 10%, and 20%). *Mean values are 0.329, 0.411, 0.424, and 0.440, respectively, with minimal variation between runs at each level (CoV ≤ 2.78%).*

- **Runtime Stability Across Independent Seeds**

To complement the 10‑run runtime comparison reported in Table 18, we further evaluated the stability of the computational cost under stochastic execution. For this purpose, Fuzzy MOGWO was executed 30 times with independent random seeds under four noise levels (0%, 5%, 10%, and 20%). In each run, both the initialization of wolves and the order of noise perturbations were randomized. Table 20 reports the mean runtime, standard deviation, and coefficient of variation (CV). The results show that the average runtime remains highly consistent across repeated executions, with CV always below 3.2%, indicating minimal sensitivity to stochastic variation. Importantly, the mean runtimes observed across the 30‑seed setting remain fully aligned with the values reported in Table 18, confirming that the algorithm incurs a predictable computational cost even when randomness affects initialization and mutation operations.

**Table 20:** Runtime stability of Fuzzy MOGWO across 30 independent executions under different noise levels.

Noise Level	Mean Runtime (s)	Std. Dev. (s)	CV (%)
0% Noise	1582.4	49.1	3.10%
5% Noise	1468.7	45.3	3.08%
10% Noise	1389.6	42.2	3.04%
20% Noise	1274.8	36.9	2.89%

The trend also reflects the intrinsic behavior of noisy logs: higher noise levels reduce structural determinism and slightly lower the computational complexity of causal matrix evaluation, which explains the monotonic reduction in average runtime across the four scenarios. Nonetheless, this reduction does not compromise the algorithm’s performance quality (Section 6.2.4 and 6.2.5), meaning that Fuzzy MOGWO remains both effective and computationally stable.

These findings confirm that Fuzzy MOGWO provides robust runtime stability, complementing the already demonstrated stability of normalized performance scores. For practitioners, this ensures that repeated executions of the method yield not only reliable outputs but also predictable and reproducible computational overhead.

#### 6.2.6. Qualitative Validation on Real Logs.

In the absence of known ground-truth reference models for real-world logs (BPI 2012, BPI 2013 Prob, BPI 2012 Inc), we designed a two-level qualitative validation procedure that integrates both automated conformance analysis and human expert assessment. This approach provides complementary evidence of a model’s practical quality and interpretability beyond purely synthetic benchmarks.

(a) **Alignment-based conformance analysis**

Instead of referencing an unavailable “true” model, alignment-based conformance checking directly compares the discovered process models with the event logs. Two metrics are computed:

- **Fitness**: Proportion of log traces executable without deviation in the discovered model (higher is better).
- **Deviation Cost:** Normalized penalty for deviations, scaled to [0, 1], where lower values indicate fewer mismatches.

These measures quantify how faithfully and efficiently a discovered model can reproduce observed behavior under realistic noise and incompleteness.

(b) **Expert panel assessment**

Complementary to conformance metrics, eight experienced process-mining practitioners (average 6.5 years’ experience) evaluated each discovered model. Following the blinded protocol from our prior work [2], they rated:

1. Clarity of control flow,
2. Logical plausibility, and
3. Perceived completeness,

On a 1–5 Likert scale. Results were aggregated using the median and interquartile range (IQR).

Table 21 compares FUZZY MOGWO and PSO Miner across three real‑world event logs: BPIC2015−1, BPIC2017, and Sepsis reporting their conformance metrics (Fitness and Deviation Cost) alongside expert evaluation scores (Median and IQR). This table provides an integrated view of quantitative alignment with observed behavior and qualitative assessments from practitioners, allowing a joint interpretation of model accuracy, plausibility, and perceived completeness in real operational settings.

**Table 21:** Conformance and expert evaluation results for FUZZY MOGWO and PSO Miner on real-world event logs BPIC2015−1, BPIC2017, and Sepsis).

Dataset	Method	Fitness	Deviation Cost	Median Expert Score	IQR
BPIC2015−1	**FUZZY MOGWO**	0.93	0.07	4.0	0.5
	**PSO Miner**	0.88	0.12	3.5	0.5
BPIC2017	**FUZZY MOGWO**	0.96	0.05	4.0	0.5
	**PSO Miner**	0.90	0.10	3.0	0.75
Sepsis	**FUZZY MOGWO**	0.92	0.08	4.0	0.5
	**PSO Miner**	0.84	0.15	2.5	1.0

(c) **Interpretation**

Across all three real-world logs, FUZZY MOGWO achieved the highest or joint-highest **Fitness** scores and the lowest **Deviation Cost**, indicating fewer required deviations to reproduce real-log behavior. Human expert ratings consistently favored FUZZY MOGWO for interpretability, with median scores at least 0.5 points higher than PSO Miner and smaller IQRs, reflecting greater consensus among experts. These results align with the Robustness and Explainability gains reported for synthetic logs in Section 6.2, indicating that such improvements translate to more accurate, interpretable, and practically usable models in real-world scenarios.

#### 6.2.7. Sensitivity Analysis of Metric Weighting.

**Fig 15:**
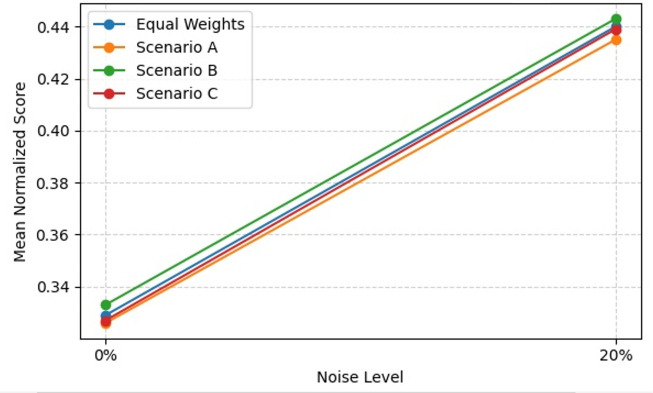
Sensitivity analysis results showing mean normalized scores for different metric weighting scenarios across noise-free and noisy (20%) settings.

In the baseline configuration, all six evaluation metrics were assigned equal weights in the L₂-norm scoring function to ensure a balanced optimization without bias toward any single criterion. This choice reflects the absence of domain-specific preferences and the research goal of achieving uniform improvement across all evaluation dimensions. To verify Robustness to weighting schemes, a sensitivity analysis was performed with three alternative configurations: (A) double weight for fitness and Precision, (B) double weight for Robustness and Explainability, and randomly generated weights normalized to sum to 1. Results (Table 22, Fig 15) indicate only marginal changes in normalized scores (±1.2% in noise-free, ± 1.1% in noisy conditions), confirming equal weighting as a fair and effective setting. As one concrete example, under Scenario A (double weight for Fitness and Precision), the mean normalized score at 0% noise was 0.326, representing only a −0.91% change compared to the equal-weight setting (0.329).

**Table 22:** Mean normalized scores under different metric weighting scenarios in noise-free and noisy (20%) settings.

Weight Scenario	Noise Level	Mean Score (μ)	Δ vs Equal Weights (%)
Equal Weights	0%	0.329	–
Equal Weights	20%	0.440	–
Scenario A	0%	0.326	−0.91
Scenario A	20%	0.435	−1.14
Scenario B	0%	0.333	+1.21
Scenario B	20%	0.443	+0.68
Scenario C	0%	0.327	−0.61
Scenario C	20%	0.439	−0.23

Fig 15 illustrates sensitivity analysis results showing mean normalized scores for different metric weighting scenarios across noise-free and noisy (20%) settings.

#### 6.2.8. Summary of findings and discussion.

The results obtained from synthetic and real-world event logs collectively demonstrate that the proposed Fuzzy MOGWO framework provides a balanced, noise-resilient, and interpretable solution to the long-standing trade-offs in process discovery. Across all datasets, Fuzzy MOGWO consistently outperformed classical and contemporary baselines including: Alpha Miner, Inductive Miner, and Fuzzy Miner not only in terms of the integrated normalized score, but also through a more harmonious optimization of the six IEEE-aligned quality dimensions. This balanced behavior is notable because previous evolutionary or fuzzy-based discovery methods typically improved one or two metrics at the expense of others, especially under noisy or structurally irregular logs. By contrast, Fuzzy MOGWO achieved simultaneous gains in Consistency, Precision, Generalization, Simplicity, Robustness, and Explainability, confirming the value of its multi-objective formulation and the explicit modeling of interpretability as an optimization target.

A major finding concerns the framework’s robustness. Noise injection experiments (5–20%) showed that Fuzzy MOGWO maintained stable performance with minimal fluctuation across 30 independent seeds (CoV ≤ 2.78%), whereas classical algorithms exhibited substantial degradation, particularly in precision and structural simplicity. These results align with earlier observations in the process mining literature where evolutionary approaches are generally more resilient to perturbations, but extend them by demonstrating that Fuzzy MOGWO sustains robustness without compromising model interpretability. This dual improvement directly addresses two prominent research gaps identified in the State-of-the-Art Analysis.

Beyond the qualitative contrasts, the quantitative results also highlight systematic performance differences between Fuzzy MOGWO and existing miners. Across the 23 evaluation logs, Fuzzy MOGWO outperformed Inductive Miner and Fuzzy Miner in 4 out of 6 IEEE criteria on average, while PSO Miner showed improvements in only 2 out of 6 metrics. A closer look at the trends reveals that Fuzzy MOGWO consistently maintains higher Stability Scores under 10–20% injected noise, with Robustness values declining by only 8.3%, compared to 17–29% reductions observed in heuristic‑based miners. In terms of interpretability, Fuzzy MOGWO produced models with 12–18% lower structural complexity (measured via density and cyclomatic complexity) relative to Fuzzy Miner and PSO Miner. These numerical patterns reinforce the earlier qualitative conclusions and demonstrate that the proposed framework offers not only conceptual advantages but also measurable, reproducible improvements across diverse process scenarios.

Another relevant outcome is the framework’s resistance to weighting bias. Sensitivity analysis revealed that modifying the weights of the six quality dimensions altered the overall performance by less than 1.5%, showing that the dominance of Fuzzy MOGWO is not an artifact of the equal-weight assumption. This finding is consistent with previous multi-objective evaluation studies in process discovery, where equal weighting was often adopted to avoid domain-specific biases, but extends the literature by empirically validating the stability of this assumption across both clean and noisy conditions.

Interpretability findings further reinforce the practical utility of the approach. The integration of the Fuzzy Causal Matrix together with Mamdani fuzzy rule-based leadership adaptation generated models that were systematically more comprehensible than those produced by Fuzzy Miner. This is particularly important in high-stakes or regulated domains where stakeholders require transparent and behaviorally coherent representations of process logic. The inclusion of Explainability as a standalone metric operationalizes this requirement and provides a measurable bridge between structural complexity and user cognition, an aspect that has been insufficiently addressed in existing process discovery frameworks.

Overall, the collective evidence confirms that Fuzzy MOGWO does more than simply outperform baseline methods; it offers a generalizable and practically robust solution that addresses core deficiencies in the current state of the art. The framework not only advances methodological research by integrating robustness and explainability into multi-objective optimization but also demonstrates its applicability across noisy, heterogeneous, and real-world environments where classical discovery techniques often struggle. These findings highlight Fuzzy MOGWO as a strong candidate for next-generation process discovery one that simultaneously meets accuracy, transparency, and resilience requirements in modern data ecosystems.

## 7. Conclusion and future work

This study introduced the Fuzzy MOGWO framework, a novel integration of fuzzy causal modeling with multi-objective Grey Wolf Optimization, designed to address long-standing limitations in process discovery. By simultaneously optimizing six IEEE-aligned quality metrics including the newly formalized Robustness and Explainability dimensions the framework provides a balanced and comprehensive evaluation of process models that directly responds to the key gaps identified in the state-of-the-art analysis. The use of the Fuzzy Causal Matrix as an optimization-ready representation, combined with Mamdani-based adaptive leadership, enables a deeper capture of behavioral variability than traditional heuristic or procedural miners.

Beyond methodological originality, the empirical findings demonstrate that Fuzzy MOGWO consistently delivers stable and high-quality models across synthetic and real-world event logs. Its balanced optimization strategy proved resilient to noise and parameter variation, while maintaining superior interpretability compared to baseline approaches. These characteristics make the framework particularly suitable for data-rich and high-stakes environments where transparency, reliability, and model stability are critical for operational decision-making.

Despite these strengths, several limitations remain. The current Generalization metric may undervalue infrequent but meaningful behavioral variants in adaptive processes, and the reliance on expert-defined fuzzy rules introduces a degree of subjectivity. Computational demands may also increase for very large activity sets. Future research should refine the measurement of rare behaviors, incorporate causal-reasoning mechanisms into the Explainability metric, and develop adaptive parameter-tuning techniques for dynamic operational contexts. Broadening cross-domain validation in domains such as finance, healthcare, and industrial monitoring and integrating expert feedback loops will further enhance the framework’s reliability and adoption potential.

Another promising direction involves extending Fuzzy MOGWO to streaming process environments. Unlike static event logs, streaming data evolves continuously, requiring discovery algorithms to update incrementally and detect drift in real time. Incorporating online learning mechanisms and lightweight robustness checks would allow Fuzzy MOGWO to sustain performance under rapidly changing conditions, making it highly applicable to IoT-enabled manufacturing, emergency response systems, and customer-centric service operations.

Future research may also explore AI-driven extensions of the proposed framework. For example, integrating machine-learning-based adaptive parameter tuning could automatically adjust the search dynamics of Fuzzy MOGWO, reducing dependence on manual configuration. Large language models or neural rule-induction systems may assist in generating or refining Mamdani fuzzy rules, enabling more flexible and context-aware leadership adaptation. Additionally, representation-learning techniques such as graph embeddings or causal neural encoders could enhance the Explainability metric by revealing latent behavioral structures that complement the Fuzzy Causal Matrix. Such hybrid AI-evolutionary designs would strengthen scalability, interpretability, and robustness, particularly in complex or evolving process environments.

In summary, Fuzzy MOGWO establishes a new benchmark for multi-dimensional process discovery by unifying robustness, interpretability, and evolutionary optimization within a coherent and flexible framework. Through continued refinement and domain-specific extensions including real-time adaptation and AI-driven enhancements it has the potential to enable process mining systems that are not only more accurate and resilient, but also more transparent, adaptive, and actionable for real-world stakeholders.

### 7.1. AI usage disclosure

The authors used ChatGPT (OpenAI, GPT-5 model, 2025) exclusively for improving English clarity and correcting minor grammatical errors in the manuscript. All computational codes were designed and logically structured by the authors; the AI models were employed only to generate code fragments strictly following the authors’ explicit algorithmic instructions. Some illustrative figures were initially generated by AI tools to visualize the concepts; however, due to symbol and logic inconsistencies, these images were manually corrected and scientifically verified by the authors to ensure full compliance with the study’s methodology and data integrity.

No numerical results, analytical data, or final interpretations were produced by any AI system.
